# Global elective breast- and colorectal cancer surgery performance backlogs, attributable mortality and implemented health system responses during the COVID-19 pandemic: A scoping review

**DOI:** 10.1371/journal.pgph.0001413

**Published:** 2023-04-04

**Authors:** Sonia Haribhai, Komal Bhatia, Maryam Shahmanesh

**Affiliations:** 1 Institute for Global Health, University College London, London, United Kingdom; 2 Africa Health Research Institute, Durban, South Africa; Indian School of Business, INDIA; Indian School of Business, INDIA

## Abstract

Globally, 28.4 million non-emergent (‘elective’) surgical procedures have been deferred during the COVID-19 pandemic. This study evaluated the impact of the COVID-19 pandemic on elective breast- or colorectal cancer (CRC) procedure backlogs and attributable mortality, globally. Further, we evaluated the interaction between procedure deferrals and health systems, internationally. Relevant articles from any country, published between December 2019–24 November 2022, were identified through searches of online databases (MEDLINE, EMBASE) and by examining the reference lists of retrieved articles. We organised health system-related findings thematically per the *Structures-Processes-Outcomes* conceptual model by Donabedian (1966). Of 337 identified articles, we included 50. Eleven (22.0%) were reviews. The majority of included studies originated from high-income countries (n = 38, 76.0%). An ecological, modelling study elucidated that global 12-week procedure cancellation rates ranged from 68.3%–73%; Europe and Central Asia accounted for the majority of cancellations (n = 8,430,348) and sub-Saharan Africa contributed the least (n = 520,459). The percentage reduction in global, institutional elective breast cancer surgery activity ranged from 5.68%–16.5%. For CRC, this ranged from 0%–70.9%. Significant evidence is presented on how insufficient pandemic preparedness necessitated procedure deferrals, internationally. We also outlined ancillary determinants of delayed surgery (e.g., patient-specific factors). The following global health system response themes are presented: *Structural* changes (i.e., hospital re-organisation), *Process*-related changes (i.e., adapted healthcare provision) and the utilisation of *Outcomes* (i.e., SARS-CoV-2 infection incidence among patients or healthcare personnel, postoperative pulmonary complication incidence, hospital readmission, length of hospital stay and tumour staging) as indicators of health system response efficacy. Evidence on procedure backlogs and attributable mortality was limited, partly due to insufficient, real-time surveillance of cancer outcomes, internationally. Elective surgery activity has decreased and cancer services have adapted rapidly, worldwide. Further research is needed to understand the impact of COVID-19 on cancer mortality and the efficacy of health system mitigation measures, globally.

## Introduction

The unprecedented coronavirus disease-2019 (COVID-19) global pandemic has disrupted lives, health systems and economies worldwide [1]. Severe Acute Respiratory Syndrome Coronavirus-2 (SARS-CoV-2) was first identified in Wuhan, China, in December 2019. Over 500 million infections and >6.3 million fatalities have been documented worldwide [2]. Despite the existence of at least 9 World Health Organization (WHO)-approved vaccines, partly as a result of inequitable distribution, the pandemic continues to severely impact health services, globally [3, 4]. Care-seeking hesitancy–attributable partly to fears surrounding contagion–has been described [5]. To prevent peri-operative SARS-CoV-2 transmission among personnel and susceptible patients, many healthcare facilities have suspended non-emergent (‘elective’) surgery services, worldwide [6, 7]. ‘Elective surgery’ constitutes any procedure, scheduled in advance and performed by a surgeon with local, regional or general anaesthesia, where delays in such intervention would not result in imminent mortality or severe morbidity [8, 9]. Global suspensions of elective surgery services have generally aligned with accredited, international public health guidelines and have been instituted to preserve resources (e.g., personal protective equipment [PPE]) and redeploy healthcare personnel (HCP) to the frontline pandemic response [10, 11].

### Cancer

Epidemiology, biomedical aspects and implications for affected patients during COVID-19 (S1 Text) Cancer is the leading cause of mortality worldwide [12]. In 2020, breast (n = 2.26 million) and colorectal cancer (CRC) (n = 1.93 million) had the first and third highest global cancer incidence, respectively [12]. Breast cancer was the most prevalent cancer type with 7.8 million females affected worldwide [13]. In 2019, globally, breast cancer-attributable disability-adjusted life-years (DALYs) amounted to 20.6 million (95% Confidence Interval [CI], 19 million– 22.1 million) and 49% of affected patients were between 50–69 years of age [14]. Approximately 2 million incident cases were documented in 2019, with the majority originating from countries with a high sociodemographic index (SDI) [14].

In terms of colorectal cancer, in 2017, 1.8 million (95% CI, 1.8–1.9 million) incident cases and 896,000 (95% CI, 876,000–916,000) fatalities were documented worldwide, accounting for 19 million (95% CI, 18.5–19.5 million) DALYs, globally [15]. The disease is less common in low-SDI countries, consistent with a lower regional prevalence of risk factors (e.g., obesity) [16]. The highest survival rates are associated with non-metastatic disease [17].

These epidemiologic patterns highlight the substantial contribution of breast- and colorectal cancer to the global burden of disease and their association with suboptimal morbidity and mortality outcomes. To prevent progression to metastasis and prolong survival, timely treatment is essential.

Generally, detailed treatment algorithms for breast- or colorectal cancer are individualised to patient needs and actioned by multidisciplinary teams, with shared decision-making by patients [18]. Biomedical treatment modalities may be classified as operative (surgical) or non-operative (medical) [19]. Broadly, operative intervention involves surgery; whereas, non-operative treatment may encompass radiotherapy or systemic agents, such as chemotherapy, immunotherapy or targeted therapy [13, 17]. Clinical management plans for breast- and colorectal cancer, respectively, may combine operative and non-operative modalities. To achieve tumour shrinkage pre-operatively, neoadjuvant therapy (notably chemoradiotherapy [NACRT]) is commonly administered [19]. Postoperatively, adjuvant chemoradiotherapy may be indicated for tumour regression, improved survival and prevention of tumour recurrence [20]. For metastatic breast- or colorectal cancer, quality of life optimisation is generally among the primary objectives of palliative therapy, which may entail medical and/or surgical intervention [17, 19]. During the COVID-19 pandemic, there has been increasing reliance on neoadjuvant therapy as an interim treatment measure until elective surgery may be safely performed [7, 21]. Early on during the pandemic, to mitigate potential, airborne SARS-CoV-2 transmission, internationally, elective laparoscopic procedures (including for the indication of colorectal cancer) were generally deferred, as some may involve gaseous insufflation of the abdomen intraoperatively [17, 22]. In selected patients with colorectal cancer, pre-operative neoadjuvant chemotherapy may be planned; subsequently, a conservative, “watch-and-wait” approach may be employed to elicit whether spontaneous tumour regression arises, negating the need for surgery [17]. To decrease the demand for surgery during the COVID-19 pandemic, where appropriate, clinicians have increasingly favoured this treatment approach [23].

Patients with cancer represent a clinically vulnerable population subgroup with the potential for disease progression and consequent inoperability [24]. Immunosuppressive chemotherapy is known to worsen their risk profile for infection, including with SARS-CoV-2, and attributable mortality [24]. The COVID-19 pandemic has significantly curtailed elective surgery service delivery for colon-, rectal and breast cancer [25, 26]. Globally, between 23 January–8 April 2020, it is estimated that >2.3 million elective procedures were deferred weekly [27]. In the U.K., by April 2021, at least 387,885 patients had been awaiting routine elective surgery for >52 weeks [28, 29]. Psychologic distress, quality-of-life impediments and incomplete care have ensued among affected patients [30]. In the U.K. alone, an estimated £2 billion is needed to ease existing case performance backlogs and generally, upper middle-income countries (UMICs) are projected to sustain the highest burden of procedure cancellations [27]. To mitigate the adverse impact of COVID-19 on cancer outcomes, various health system responses (HSRs), such as the establishment of ‘COVID-19-protected’ hospital pathways (i.e., designated healthcare units for patients without SARS-CoV-2 infection, separate to those with the condition), have been proposed [22, 31, 32].

### Focus areas and study rationale: Breast and colorectal cancer

In this scoping literature review, we aimed to evaluate the global impact of elective surgery service suspensions, as defined by accrued surgical backlogs and attributable mortality, for adults (age ≥18 years) with breast- or colorectal cancer during the COVID-19 pandemic. We also evaluated the interaction between health systems and procedure deferrals, internationally. Significant evidence is presented on how insufficient pandemic preparedness necessitated a decrease in elective cancer surgery activity within health systems, globally. However, as discussed below, sufficient data is not yet available on the impact of deferred elective breast- or colorectal oncology procedures on clinical outcomes (notably, mortality) during the COVID-19 global pandemic.

We focused on the abovementioned cancer types due to their high global incidence and prevalence and because surgical treatment is known to improve clinical outcomes (e.g., prognosis) [12, 33, 34].

## Methods

The population, exposure, comparator and outcome of interest are tabulated (S1 Table and S2 Text).

### Article selection and data extraction

We executed a systematic literature search on 24 November 2022 using the MEDLINE and EMBASE databases. A librarian (HC) approved the final search strategy, including the primary search domains and implemented medical subject headings (MeSH) (S2 and S3 Tables). We expanded our search by examining the reference lists of retrieved articles (i.e., snowball method).

We pre-developed a data extraction form and updated this iteratively. Data items included article characteristics (e.g., study design), primary outcomes and health system-related findings (S1 Data). We searched the databases and exported the search results to the form. We resolved uncertainty regarding article selection and data extraction through discussion among co-authors. Applying specific eligibility criteria (Table 1), we initially screened titles and abstracts, followed by full-length articles. We removed duplicates and grouped studies according to the outcomes of ‘impact of COVID-19’ (i.e., surgical backlogs or mortality) or ‘implemented health system responses’ or both. We created sequential, dated duplicates of the populated data form for storage and reference. We assessed the quality of individual studies using *Critical Appraisal Skills Programme* (CASP) checklists [35].

**Table 1:** Eligibility criteria and rationale.

No.	Inclusion criteria:	Rationale:
1.	• English-language publications (only)	• Limited access to translation services
2.	• Published between December 2019 and 24 November 2022	• Timeline corresponds with first identification of SARS-CoV-2 in December 2019, in Wuhan, China
3.	• Country-of-origin: Any	• For comprehensive appraisal of evidence on global outcomes
4.	• Pre-prints (not excluded)	• For comprehensive appraisal of evidence • Evidence is emerging contemporaneously on this current global health challenge
5.	Scope: • Elective breast- or colorectal cancer surgery backlogs, attribu mortality and/or implemented health system responses during the COVID-19 pandemic	• To synthesise known findings and, identify existing gaps in the evidence base • To address the research aims
6.	Data synthesis on surgical case performance backlogs: • Articles based on global and/or institution-level reductions in surgical activity (i.e., number of elective procedures performed) during the COVID-19 pandemic, as compared to surgical activity pre-COVID-19	• Included due to a prevalent scarcity of evidence on absolute backlogs outcomes • Global and institution-level reductions in surgical activity would, by reasonable inference, contribute to overall case performance backlogs
**No.**	**Exclusion criteria**	**Rationale**
	• Non-English language publications (Unable to locate translated version)	• Limited access to translation services
	• Unable to locate full-text article	• Avoid potential bias from incomplete appraisal of study findings
	• Significant methodological quality deficit	• Findings insufficiently reliable
	• Opinion pieces, correspondence articles (apart from research letters) • Guidelines on operative aspects • Protocols, posters • Conference abstracts • Symposium minutes	• Opinion pieces–Findings subjective • Operative guidelines–Limited relevance to research question, specialist operative techniques fall beyond the scope of this review • Other–Definitive, complete evidence prioritised for review
	Out of scope: • Reported on outcomes for other diagnoses (apart from breast- or colorectal cancer, in isolation)	• Small-scale review, hence approximately 1,288 articles on impact for other cancer types excluded • Other conditions not relevant to research question
	Out of scope: • Focused on emergent surgery, without reporting on outcomes for elective surgery in isolation	• To avoid misclassification bias between outcomes for emergent surgery and those for elective surgery

### Conceptual model

For health system responses, we synthesised findings thematically with reference to a conceptual model by Donabedian (1966), outlined below [36].

Donabedian (1966) published a *Structures-Processes-Outcomes* model for urgent healthcare quality evaluation [36]. The model excludes economic considerations and proposes that within health systems, *structures*, in tandem with *processes*, determine health *outcomes* [36]. The model has been criticised as technocratic; however, its relevance persists, as it synthesises some practical, core components of optimal health system functioning [37].

*Structures* pertain to the “adequacy” and availability of infrastructure and equipment, health policy, data systems, evidence-based clinical guidelines and providers’ medical accreditation–i.e., the “settings and instrumentalities” with which high-quality healthcare provision would presumably follow [36]. Generally, these elements are easily measurable and observable [36].

*Processes* entail healthcare provision at the level of provider-patient interactions and exclude community-based initiatives [36]. This domain deals with the “appropriateness, completeness…justification…technical competence[,]” continuity and acceptability with which healthcare is rendered [36]. Quality evaluations at the level of *processes* are “less stable and less final” than those involving the measurement of *outcomes* [36].

*Outcomes* are indicators of “recovery, restoration of function and [survival]” (e.g., mortality and vaccination rates); usually, they can be precisely measured and widespread consensus exists regarding their value in optimising population health [36]. “[F]actors other than medical care” may confound exposure-outcome associations; to mitigate spurious quality-of-healthcare appraisals, the model thus proposes “comparative studies of outcomes, under controlled situations” (e.g., randomised controlled trials) [36].

A limitation of the model is that it does not characterise the independent relationship between *structures* and *outcomes* or, between *processes* and *outcomes* [36].

## Results

**Fig 1:**
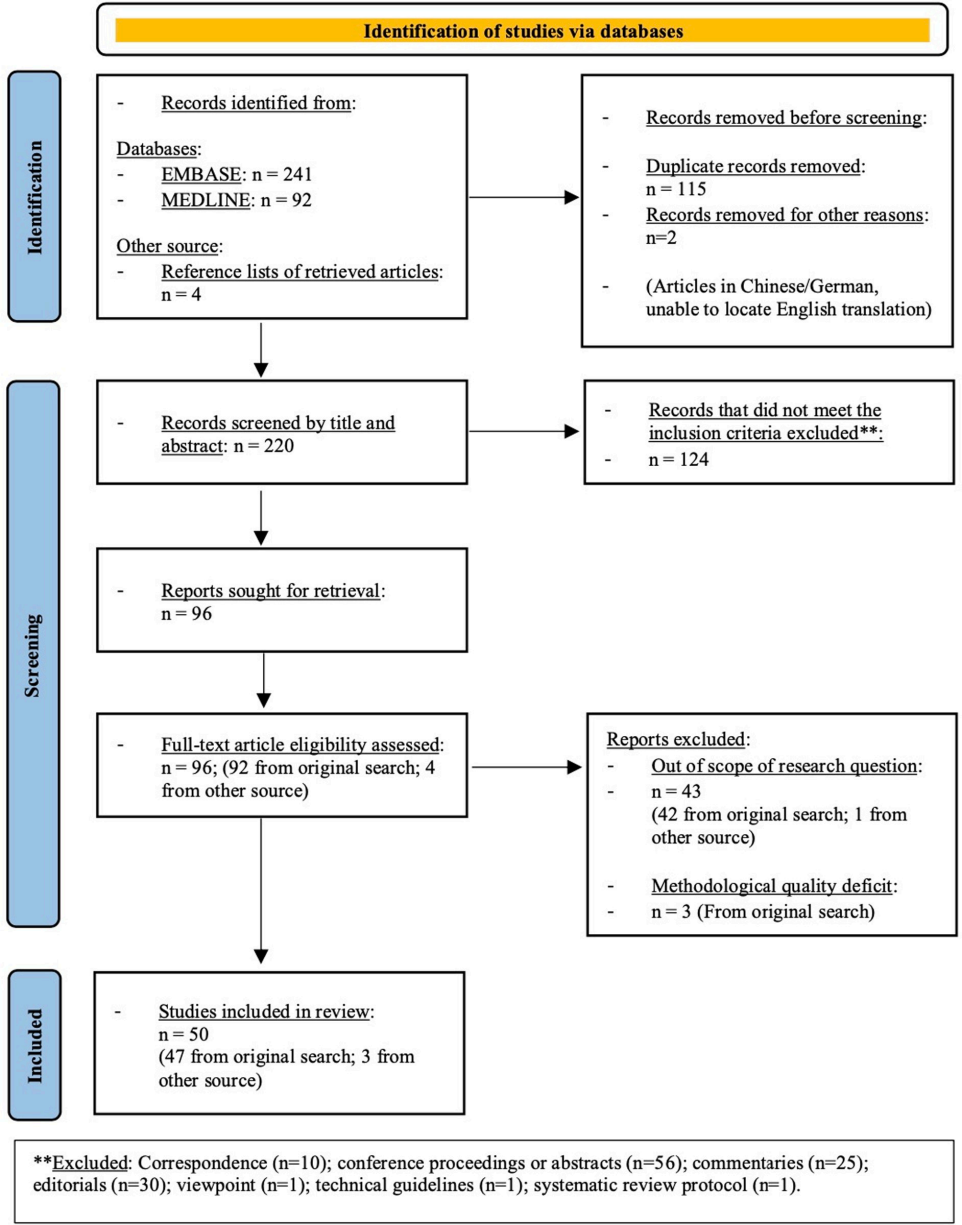
Preferred Reporting Items for Systematic Reviews and Meta-Analyses (PRISMA) flow-diagram of article identification process [76].

The final review comprised 39 primary research articles (20 case series, 7 cross-sectional studies, 4 cohort studies, 1 case-control study, 4 ecologic studies, 2 research letters and 1 qualitative study,) and 11 reviews (including 2 systematic reviews) (Fig 1) [5–7, 9, 18, 21–27, 38–75]. At the time of review, two of the included studies were preprints [57, 74]. Most of the included articles were from high-income countries (HICs) (i.e., n = 38, 76.0% from HICs and n = 12, 24.0% from low- and middle-income countries [LMICs]). Seventeen studies reported on the ‘impact of COVID-19’ [25, 27, 44, 47, 48, 50–52, 57, 68–73, 75], 30 reported on ‘health system responses, effects or challenges’ [6, 7, 18, 21–24, 38, 40–43, 45, 46, 49, 53–56, 58–67, 74] and 3 reported on both [5, 26, 39]. We present the key findings below in a narrative synthesis, according to the primary outcomes (S1 Checklist).

### Mortality

None of the primary research studies reported on mortality attributable to delayed surgical intervention during COVID-19. However, one systematic review by Whittaker et al (2021) reported the overall survival and disease-free survival among patients affected by prolonged time-to-surgery, following a diagnosis of colorectal cancer; the authors included 7 studies in a meta-analysis [25]. Three of the 7 included studies, encompassing results from 314,560 patients, revealed that extended delays to elective colorectal cancer surgery led to reduced overall survival and disease-free survival [25]. At 4 weeks’ delay, for overall survival, the hazard ratio (HR) for 6 datasets was 1.13 (95% CI, 1.02–1.26, *p* = 0.02); whereas, at 12 weeks’ delay, the pooled HR for 3 datasets was 1.57 (95% CI, 1.16–2.12, *p* = 0.004) [25]. The ‘number needed to harm’ for a 4-week or 12-week delay was 35 and 10, respectively [25]. The authors concluded that extended (>4 weeks) delays to elective colorectal cancer surgery are associated with increased mortality and recommended using clinical risk assessments to prioritise case performance [25] (S4 Table).

### Backlogs

An ecological study reported that the true magnitude of global, pandemic-attributable elective surgery cancellations “remain[s] unquantified” [27]. However, by mathematical modelling, it was projected that between 23 January–8 April 2020, across 190 countries, >28.4 million elective surgical cases were deferred [27]. Approximately 37.7% (n = 2.3 million of 6.1 million cases) of elective oncologic procedures were estimated to have been postponed; this predicted a median of 45 weeks for procedural backlog clearance, assuming a 20% increase in routine surgery performance [27]. In terms of colorectal cancer, approximately 35.9% (n = 486,583 of 1.3 million) of the normal case volume (at full capacity) was cancelled [27]. Because breast surgery for benign indications was estimated to contribute to <5% of the global procedural backlog, cases were pooled into an ‘Other surgery’ category, for which a 12-week cancellation rate of 81.7% (i.e., n = 6.7 million of 8.2 million) was projected [27]. Stratified by World Bank income groups, global 12-week cancellation rates range from 68.3%–73%; Europe and Central Asia accounted for the highest number of cancellations (n = 8.4 million) and sub-Saharan Africa contributed the least (n = 520,459), consistent with “low baseline surgical volume [s]” within this region [27].

No other studies quantified or estimated absolute elective breast- or colorectal cancer procedure backlogs. Further institutional elective surgery activity reduction outcomes across studies are tabulated below (Table 2).

**Table 2:** Global institutional elective procedure performance reductions and outcome determinants.

**BREAST CANCER**							
**No.:**	**Authors (year of publication)**	**Study design**	**Country**	**No. of patients, (year)**	**No. of elective procedures performed (%), (year), *p*-value**	**Case performance reduction, (%)**	**Outcome determinants (qualitative)**
1	Fregatti et al. (2020)	Cross-sectional study	Italy	n = 85	n = 71 (83.53%)	16.5%	• Pre-operative SARS-CoV-2 screening (surgery delayed if positive) • Potential information bias: Patients’ responses to coronavirus symptom screen • Healthcare personnel redeployment • Reduced availability of operating theatres
**COLORECTAL CANCER**							
2	Giuffrida et al. (2020)	Case series	Italy	n = 13	n = 13 (2020) vs. Pre-pandemic: n = 25 (2019)	48%	• Local COVID-19 prevalence • Lockdown • Conversion of hospital departments to COVID-19 units • Insufficient hospital bed capacity • Delayed screening and diagnostic tests • Increased care-seeking hesitancy and resultant delays in diagnosis • Disrupted continuity of care (daily changes of practice and workplans)
3	Tejedor et al. (2021)	Case series	Spain	n = 301	n = 259 (86.05%)	13.95%	• Limited study follow-up period • Hospital case volume of COVID-19 • Pre-operative SARS-CoV-2 screening (surgery delayed if positive) • Referral to other facilities • Eligibility for interim NACRT • PPE shortages • Loss to follow-up • Other reasons (unspecified)
4	Matosevic et al. (2021)	Case series	Croatia	n = 116	n = 116 (2020) vs. Pre-pandemic: n = 147 (2019)	21.09%	• Clinical anaesthetic risk assessment • Clinical risk profile overall (age, comorbidities, physical habitus) • Pathologic tumour staging • Eligibility for interim NACRT or ‘watch-and-wait’ approach • Delayed screening and diagnostic tests • Type of procedure required • Hospital resource availability
5	Feier et al (2022)	Case series (Comparative to pre-pandemic period)	Romania	n = 147	n = 29 (from 2020–2021) n = 68 (from 2018–2019) n = 50 (from 2016–2017)	57.3% (compared to 2018–2019) 42% (compared to 2016–2017)	• Delayed presentation: Local public health messaging to patients to present to hospital only in the instance of severe symptoms or (surgical) emergencies • Care-seeking hesitancy among patients due to fear of contagion • Delayed screening and diagnostic tests • Conversion of intensive care unit to COVID-19 unit • National ‘state of emergency’ declaration on 16 March 2020
6	Tarta et al (2022)	Case series	Romania	N = 198 n = 83 (2019) n = 80 (2020) n = 84 (2021)	n = 57 (68.7%) (2019) n = 40 (50.0%) (2020) n = 36 (42.9%) (2021) *p* = 0.002	25.8% (comparing 2021 to 2019) 7.1% (comparing 2021 to 2020)	• Suspension of elective surgery services • Suspension of screening colonoscopy services • Delayed presentation due to care-seeking hesitancy • Higher number of emergencies due to delayed presentation with resultant development of complications • Elective procedures delayed until patients demonstrated negative SARS-CoV-2 PCR test results
7	Sozutek et al. (2021)	Cohort study	Turkey	n = 99 n = 40 (Colon cancer) n = 59 (Rectal cancer)	n = 99	0%	• No SARS-CoV-2 infections (Healthcare personnel or patients) during study period • Clinical anaesthetic risk assessment • Clinical risk profile overall (age, comorbidities) • Pathologic tumour staging • Eligibility for interim NACRT • Fidelity to standardised treatment algorithms and clinical guidelines • Re-organisation of hospital units, physical distancing of beds • Hospital resource availability
8	Cui et al. (2021)	Case series (Comparative to pre-pandemic period)	China	n = 67	n = 67 (2020) vs. Pre-pandemic: n = 101 (2019) n = 104 (2018)	66.34%	• Adherence to national- and professional epidemic control standards • Decreased number of health consultations • Pathologic tumour staging • Limited bed capacity • Patient avoidance of long-distance traveling to health facilities due to fear of contagion
9	Sobrado et al. (2021)	Cross-sectional study	Brazil	n = 90 (For diagnosis of CRC)	n = 90	0%	• Pathologic tumour staging • Eligibility for interim NACRT • Decreased number of health consultations during COVID-19 pandemic • Local COVID-19 public health policy • Pre-operative SARS-CoV-2 screening test result (surgery delayed if positive) • No SARS-CoV-2 vaccine availability at the time of study
10	Santoro et al. (2021)	Cross-sectional expert survey: Expert respondents reported ‘Delays’ or ‘No delays’ in CRC diagnosis or surgery	Global (84 countries)	Total: n = 1,051 n = 745 (70.9%)–Reported ‘Delays’ n = 306 (29.1%)–Reported ‘No delays’	N/A	70.9% (Affected by delays)	• Local COVID-19 prevalence and public health response • Hospital involvement in COVID-19-care (e.g., Fully- vs. partially dedicated) • Type of hospital (academic vs. non-academic, bed capacity) • Availability of ‘COVID-19-free’ units • Availability of COVID-19 clinical protocols • Delayed diagnostic services (endoscopy, histopathology) • Suspended multidisciplinary meetings • PPE shortages • Pre-operative SARS-CoV-2 screening test result (surgery delayed if positive) • Pathologic tumour staging • Eligibility for interim NACRT • Personnel infection with SARS-CoV-2 • Personnel redeployment
**BREAST- OR COLORECTAL CANCER**							
11	Aguiar et al. (2020)	Research letter: Cross-sectional study	Brazil	Total: n = 540 Breast: n = 88 Colorectal: n = 32 Other: n = 420	n = 454 (84.1%) Breast: n = 83 (94.32%) Colorectal: Not stated (Gastrointestinal: n = 96)	Breast: 5.68% Colorectal: Not stated (Gastrointestinal: 10.42%)	• Pre-operative SARS-CoV-2 screening test result (surgery delayed if positive) • Patient-specific factors: • Voluntary cancellation of procedure • Access to health insurance • Geographic residential proximity to healthcare facilities
12	Nepogodiev et al. (2020) COVIDSurg Collaborative Study	Ecologic, modelling study	Global: 84 countries	CRC: n = 486,583 Benign breast- or ‘other’ cancer: n = 6,760,731	CRC: n = 486,583 Other diagnoses (incl. benign breast conditions): n = 6,760,731 vs. Pre-pandemic: CRC: n = 1,353,952 Other surgery incl. benign breast surgery: n = 8,273,626	CRC: 35.9% Other surgery incl. breast surgery: 81.7%	• Local COVID-19 prevalence • Local public health policy • Fidelity to national or international clinical guidelines, including with respect to suspension of elective surgery

Abbreviations–CRC: Colorectal cancer

Five case-series studies, conducted at an institutional level, compared the number of colorectal cancer procedures performed during the pandemic to that performed during preceding years, pre-COVID-19; one of the studies was from an UMIC (China) and four were from HICs (Italy, Romania, Croatia) [39, 57, 68, 70, 75]. The relative percentage reduction in the institutional volume of elective colorectal procedures performed ranged from 11.08%–57.3% across these studies [39, 57, 68, 70, 75].

Four observational studies (2 cross-sectional studies, 1 cohort- and 1 case series study) and 1 ecologic, modelling study reported the number of elective colorectal cancer- or breast- and colorectal cancer procedures completed without delay within a set period during the pandemic; 2 of these studies were from UMICs (Brazil, Turkey), 1 was from a HIC (Spain) and 2 were international studies [27, 69, 71–73]. The percentage reduction in the institutional breast cancer case performance ranged from 5.68%–16.5% [5, 27]. In terms of colorectal cancer, this percentage ranged from 0%–70.9% [69, 71–73]. Studies that reported a 0% proportionate reduction generally ascribed this to health system response efficacy and fidelity to standardised treatment algorithms [71, 72].

The results of three studies of multi-center elective surgical performance reductions [44, 47, 48] and three ecological studies of elective surgical case performance rates [50–52], respectively, are tabulated (Tables 3 and 4).

**Table 3:** Global multi-center elective procedure performance reductions and outcome determinants.

COLORECTAL CANCER							
No.	Authors (Year of publication)	Study design	Country	No. of healthcare centers (n) (Additional details)	Study period	Case performance reduction (%) (Additional details)	Outcome determinants
1.	Hunger et al (2022)	Case-control	Germany	66 (Nationwide; n = 176,783 patients)	Period 1: March 2019–February 2020 Period 2: March 2020–February 2021	9.0% (*p* = 0.002)	• Ministry of Health recommendation to suspend elective surgical services (March 2020) • Low local COVID-19 prevalence • No uniform national pandemic response • Seasonal effect: Study period spanned seasons when regional COVID-19 incidence was lower, allowing for immediate surgery rescheduling • Oncologic procedures prioritised • Data were from medium-sized centers only • Limitations of available data (incomplete) • Delayed care-seeking due to fear of contagion
2.	de la Portilla de Juan et al (2021)	Cross-sectional survey	Spain	67 (Nationwide)	February–April 2020	79.1% (Complete or partial cessation of surgery) 32.8% (Complete cessation, 22/67 centers) 46.3% (Partial cessation, 31/67 centers)	• Eligibility for interim NACRT (patient-specific) • Non-uniform mitigation capacity across centers • Non-uniform COVID-19 prevalence nationwide • Institutional adaptation of clinical protocols • ‘Window of opportunity’ approach–Elective surgery performed whenever feasible relative to local COVID-19 upsurges • National “state of alarm” declaration
3.	Purdy et al (2022)	Cross-sectional	USA	559 (Data obtained from Vizient Database; n = 5,605 patients with CRC)	November 2019–June 2020	39.0% (*p*<0.001) (Procedure type: Colectomy)	• No availability of SARS-CoV-2 test kits during initial months of pandemic, unable to proceed with surgery among untested patients • Recommendation from local Surgeon General to limit elective surgery performance during COVID-19 (March 2020) • Delayed diagnosis: Screening and diagnostic testing (e.g., colonoscopy) suspended • Patient-specific factors: • Patients’ comorbidities precluded hospital admission during COVID-19 (high risk profile) • Delayed care-seeking due to fear of contagion

**Table 4:** Global ecological studies of elective procedure performance rates and outcome determinants.

BREAST CANCER							
No.	Authors (Year of publication)	Country	Study data source	Study period (additional details)	Case performance (%), additional details	Procedure	Outcome determinants
1.	Rubenstein et al (2022)	USA	American College of Surgeons National Quality Improvement Program	• 2019–2020	• 10.7% reduction	• Breast lumpectomy or mastectomy with or without reconstruction • Prophylactic contralateral mastectomy	• Prevailing local public health guidelines restricted the performance of breast reconstruction surgery and preventative mastectomies during COVID-19
**COLORECTAL CANCER**							
2.	Eklov et al (2022)	Sweden	Swedish Colorectal Cancer Registry	• 2019–2020	• 4% increase, *p*<0.01 • Performance:) 54% (2019) 58% (2020)	• Laparoscopic procedures for colorectal cancer	• Limited local impact of COVID-19 on colorectal cancer service delivery
3.	Meijer et al (2022)	Netherlands	Netherlands Cancer Registry	• March–May 2020 (First national peak of SARS-CoV-2 • 2018–2019	• 3% reduction • Performance: 88% (2020) 91% (2018) 91% (2019)	• Elective procedures for colon cancer	• Minimal disruptions to health services during first, national SARS-CoV-2 peak

Eleven of the 18 studies that reported on elective surgery activity were undertaken at an institutional level, which limited their statistical power to show a difference [5, 26, 39, 57, 68–73, 75]. Case performance reductions were not caused solely by elective surgery service suspensions; ancillary pandemic- or patient-specific determinants are tabulated (Table 5).

**Table 5:** Ancillary determinants of elective breast- and colorectal cancer surgery delays, apart from service suspensions.

**PUBLIC HEALTH DETERMINANTS**
• Epidemiologic and public health policy considerations: Local COVID-19 prevalence Nationwide lockdown Declaration of national state of emergency Public health messaging to present to hospital only in case of severe symptoms or emergencies Suspension of elective screening- and diagnostic testing services • Access to COVID-19 vaccines not yet established in region or country location
**INSUFFICIENT HEALTH INFRASTRUCTURE OR PANDEMIC PREPAREDNESS**
• Resource limitations: Limited availability of COVID-19-adapted clinical guidelines Reduction in number of operational operating theatres, to prevent contamination Limited availability of ‘COVID-19-free’ units Limited availability of smoke filtration equipment for laparoscopy Limited bed capacity due to physical distancing Shortage of PPE, hospital beds, SARS-CoV-2 test kits Reduction in health workforce capacity due to SARS-CoV-2 infection and/or redeployment of healthcare personnel
**DISRUPTED SERVICE PROVISION PATHWAYS**
• Positive preoperative SARS-CoV-2 test: (Patient) Admission to COVID-19 unit or 14-day-, home-based self-isolation with repeat, post-convalescence SARS-CoV-2 testing • Slowed service delivery: Requirement for pre-operative, home-based quarantine for defined period (e.g., 14 days) if travelling from out-of-state to undergo surgery at healthcare facility Increased time requirement for sanitisation and ventilation of operating theatres between consecutive procedures • Type of procedure required: Elective breast reconstruction procedures deferred Procedures with high operative time requirement deferred • Disrupted continuity of care: Suspension of multidisciplinary team meetings Decreased frequency of in-person health consultations Diversion of patients to alternative healthcare facilities NACRT halted during regional COVID-19 surges (to minimise immunocompromise)
**CASE PRIORITISATION REQUIREMENTS**
• Patient unfit for surgery: Anaesthetic or overall clinical risk profile (age, comorbidities) SARS-CoV-2 infection detected pre-operatively • Case prioritisation per assessment: Staging, grading and tumour characteristics Eligibility for interim medical therapy Risk of any medical or surgical complications secondary to delayed surgery
**PATIENT-SPECIFIC FACTORS**
• Social determinants of health: Access to health insurance Residential proximity to health facilities • Individual factors: Care-seeking hesitancy due to fears of contagion Uncertainty regarding navigation of adapted health system pathways Loss of confidence in healthcare provider or health system, change to alternative healthcare provider Decreased acceptability of health service delivery via telehealth platforms Declined consent for surgery Non-adherence to (non-operative) medical treatment Referral to alternative healthcare facilities Loss to follow-up

**Global health system responses** (S4–S14 Tables)

**Fig 2:**
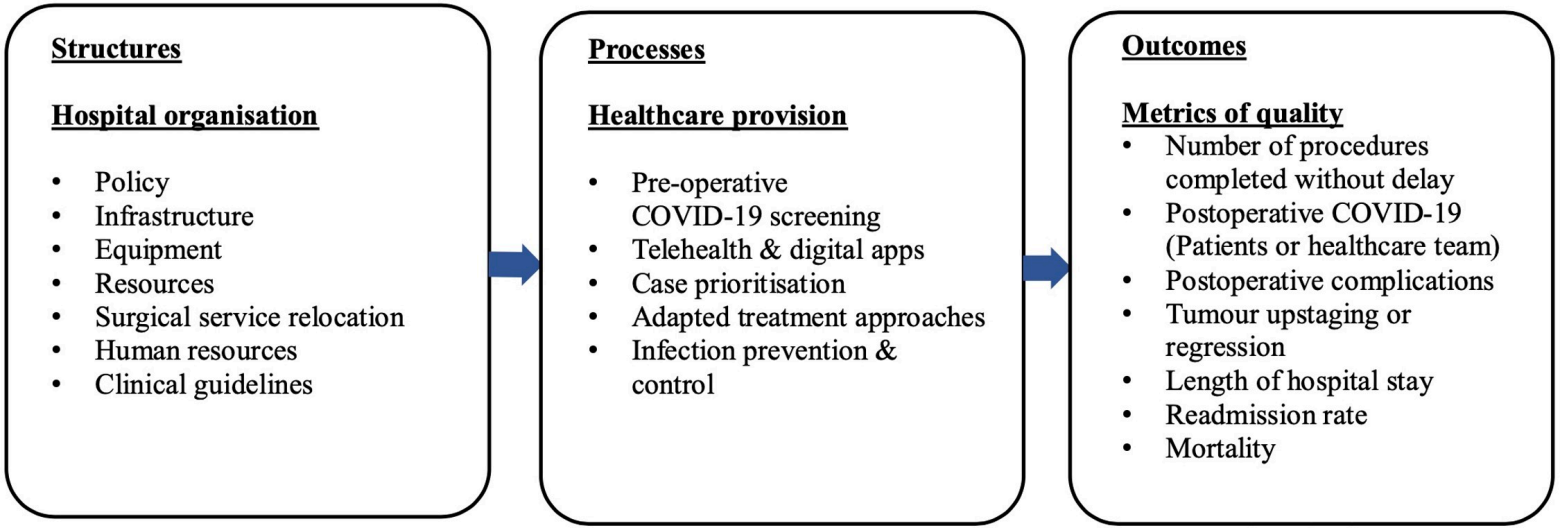
Key, global health system response themes with reference to the *Structures-Processes-Outcomes* model by Donabedian (1966) [36].

Nine of 23 articles on health system responses pertained to breast cancer; 8 to colorectal cancer and 6 to breast- and colorectal cancer [5–7, 18, 21–24, 26, 38–42, 43, 45, 46, 49, 53–56, 74]. Key themes are presented narratively with reference to the *Structures-Processes-Outcomes* model by Donabedian (1966) [36] (Fig 2).

***Structures*** (S5–S10 Tables)

Twenty-three articles reported structural health system responses; 17 originated from HICs (Italy, France, U.K. and USA) and 6 from LMICs (Brazil, India, China, Turkey) [5–7, 18, 21–24, 26, 38, 41, 43, 53–55, 61–67, 74]. The studies each outlined institutional health system responses, with exceptions of a global cross-sectional survey, a cohort study, a systematic review and a scoping review, which respectively synthesised data from international sources [55, 58–60].

### Hospital organisation

#### Policy

Italian and French studies respectively described an institutional policy to proceed with elective procedures for oncologic indications [6, 7, 21]. Eight studies reported a prohibition on hospital visitation [6, 21, 22, 26, 61, 63, 64, 66]. One of these studies described telephonic updates to consenting patients’ relatives, provided by the Surgery Unit Chief [21]. At Italian centres, patients with suspected SARS-CoV-2 infection would be isolated within single rooms, separate from pre-hospitalisation waiting areas where public access was restricted [21, 61]. To minimise absenteeism among healthcare personnel, vaccination against influenza and the utilisation of facemasks were mandated at a Brazilian institution, in line with local Ministry of Health regulations [18].

#### Infrastructure, equipment and resources

Thirteen studies (originating from Italy, U.K., France, Brazil, India and China, respectively) described the establishment of designated COVID-19 screening-, diagnostic or treatment pathways, separate to other hospital departments for patients without COVID-19 [6, 7, 18, 22, 24, 41, 53, 59, 62–64, 66, 67]. To aid patients’ navigation of adapted hospital pathways, a hospital map was designed at an Italian centre [6].

A separate U.K. study reported the maintenance of a database to record information on all surgical patients [22].

Multiple studies documented the establishment of SARS-CoV-2 screening facilities for patients, including symptom screens or, in Chinese and U.K. contexts, pre-operative thoracic computed tomography (CT) [6, 21, 62, 66].

To prevent contamination, a French institution decreased the number of operational operating theatres (OTs) from 20 to 5; additionally, 6 intensive care units were designated as COVID-19 units [7]. Hospital beds were physically distanced, which had the implication of reduced overall bed capacity within U.K., Italian and Chinese settings [22, 58, 59, 61, 62, 66].

An adequate interval between consecutive procedures for thorough OT sanitisation and negative-pressure OT ventilation, including a minimum of 20–25 hourly air exchanges, was maintained in China and Italy, respectively, along with the opening of windows for enhanced, natural ventilation [23, 60, 62].

To mitigate SARS-CoV-2 aerosolisation, smoke extraction- and air filtering devices were used during laparoscopy within Italian and U.K. settings [6, 22, 23, 60, 67]. To prevent contamination, anaesthetic soda lime was disposed and replaced after every procedure [60].

Two studies–from India and China, respectively–emphasised safe waste disposal [24, 62]. Four studies further highlighted the utilisation of air disinfectants and/or the meticulous sanitisation of hospital surfaces, elevators, wards, OTs and ‘COVID-19-protected’ units [22, 60, 62, 66].

Adequate stockage and preservation of PPE, especially for high-risk procedures, was widely recommended [7, 18, 24, 26, 58, 60, 65]. Demarcated areas for ‘donning and doffing’ were erected at U.K.-based and Italian institutions [6, 63]. Lastly, one study discussed ensuring an ongoing supply of oncologic drugs, considering an increased reliance on non-surgical therapy at an Indian institution [64].

#### Relocation of elective surgery services

In the U.K., 4 of 16 hospitals (across 8 regions) outsourced elective colorectal cancer procedures from the public- to the independent (private) sector [74]. Associated challenges included unfamiliarity among healthcare personnel working in the public sector with the policies of the independent sector, variation in the intersectoral thresholds for surgery and “restrictive criteria” for acceptance of patient referrals to the independent sector [74].

#### Clinical practice guidelines

To standardise healthcare provision, a Brazilian institution formulated diagnostic clinical criteria for COVID-19 [18]. To monitor the regional COVID-19 prevalence, oversee health system responses and adapt international guidelines to local practices, a multidisciplinary crisis committee was assembled [18]. Similarly, in a U.S. setting, guidelines for clinical case prioritisation were continuously adapted in response to the pandemic [65].

In a Turkish context, there was a drive to expedite (pre-print) research publications, to the end of global collaboration in developing rapid, evidence-based solutions for continued healthcare delivery during COVID-19 [54].

#### Human resources

Multidisciplinary collaboration for the design of health system responses was documented at Italian, French, U.K., Brazilian and Chinese institutions, respectively [7, 18, 21, 59, 61, 62]. Moletta et al (2020) further described assigning clinical case prioritisation to more experienced clinicians [60].

At Chinese and Italian centres, healthcare personnel were screened for SARS-CoV-2; this included temperature checks 3 times daily, 4-hourly facemask changes, self-reported symptom screens, documentation of close contact with individuals with COVID-19 and fortnightly SARS-CoV-2 testing [6, 62]. At one Italian centre, healthcare personnel underwent serum immunoglobulin screening for SARS-CoV-2 [61]. At a U.K.-based institution, healthcare personnel were advised to minimise contact with patients with SARS-CoV-2 infection for 7 days before entering surgical units and to self-isolate for 48 hours before surgical shifts [22].

Food supplies were handed to staff of ‘COVID-19-protected’ units within air-locked corridors and the containers sanitised before delivery to patients [22].

Internationally, to maximise efficiency, more experienced surgeons were assigned to perform procedures and independent (single clinician) ward rounds [7, 22, 60, 66]. At a Brazilian centre, non-anaesthetic personnel were excluded from OTs during high-risk, potentially aerosolising airway management procedures [18]. In a French setting, airway management procedures were restricted to anaesthetic specialists [7]. Within Italian and global contexts, intraoperative traffic and the presence of healthcare personnel within OTs was minimised [6, 18, 59, 61]. At a U.K.-based centre, restrictions were applied to the maximum number of procedures performed daily [22]. For decontamination, a post-procedure shower and change of scrubs for surgeons was recommended [60].

At an Italian institution, personnel were deployed specifically to assist patients with cognitive or physical disability, considering hospital prohibitions on public visitation [21].

In the U.K., a qualitative study with 27 interviewees (22 surgeons, 3 colorectal nurse specialists, 1 stoma nurse, and 1 gastroenterologist) from 16 hospitals across 8 regions, reported the redeployment of healthcare personnel to COVID-19 care units [74]. Resultant delays in the performance of elective surgery, ranging from 0–12 weeks in duration and dependent on varying inter-hospital pandemic response capacity, were described [74].

Across European institutions, hospital personnel were trained on hygiene measures; nurses, specifically, were trained to implement other mitigation measures, service dedicated COVID-19 units and provide home-based healthcare postoperatively [6, 7, 21, 26, 63]. Training on the diagnosis and management of (postoperative) COVID-19 has also been emphasised [55].

At an Indian institution, to facilitate coping, mental wellbeing support services were made available for healthcare personnel [43].

***Processes*** (S11 and S12 Tables)

### Healthcare provision

#### Pre-operative SARS-CoV-2 screening

Pre-admission SARS-CoV-2 screening for patients was widely operationalised; this generally comprised a symptom screen, nasopharyngeal swab- or serum immunoglobulin testing and referral of patients with possible COVID-19 to designated care units with elective surgery postponement by 14–21 days, or until patients were able to demonstrate a negative (non-reactive) result on diagnostic re-testing for SARS-CoV-2 infection, post-convalescence [5–7, 18, 21, 23, 24, 26, 53, 58–67]. Within Italian and Brazilian settings, telephonic symptom screens were conducted [7, 18, 21]. At other institutions in Italy, U.K. and China, thoracic CT was employed [7, 53, 62, 66]. At a Brazilian centre, to incentivize patient adherence, pre-operative SARS-CoV-2 screening was performed at no additional cost to patients [5]. In a U.S. context, out-of-state patients, travelling to access elective surgery, completed a mandatory, pre-operative 14-day quarantine [65]. In the U.K., to prevent possible healthcare facility-level outbreaks of (community-acquired) SARS-COV-2, patients requested their household contacts to adhere to a 2-week quarantine pre- and post-procedure [63]. The approach of many institutions was to consider all presenting patients as individuals with SARS-CoV-2 infection unless proven otherwise [60]. For confirmed cases, disease notification and home-based self-isolation with post-convalescence repeat SARS-CoV-2 testing, were instituted [21, 26, 62].

#### Telehealth and digital apps

Multiple studies described the utilisation of telehealth and digital apps (e.g., for pre-admission triage, healthcare provider-patient consultations, multidisciplinary teleconferences and communication surrounding adapted health system pathways) [18, 21, 23, 26, 58, 59, 61, 62, 64, 67].

#### Case prioritisation

French and U.K. studies respectively described basing surgical case prioritisation on tumour staging; the frequency of in-person follow-up was decreased for patients in stable remission [7, 67]. Another study outlined a similar approach; however, patients’ holistic clinical risk profiles, including their eligibility for pre-operative, interim medical therapy, was considered and breast reconstruction procedures were generally deprioritised [64]. An Italian institution prioritised elective procedures per individual patients’ clinical risk profile for medical complications [21]. In U.S. and U.K settings, surgical cases were prioritised per the maximum possible delay-to-surgery that could be sustained without a risk of major medical or surgical complications [65, 66].

#### Adapted treatment approaches

To include recommendations of less invasive, non-aerosolising tests (e.g., home-based faecal immunochemical self-testing and capsule colonoscopy) as first-line screening investigations, a Turkish institution modified its colorectal cancer screening protocols [54].

Given the unprecedented demand for health services within an Indian setting, a revision of standard treatment protocols for patients with cancer and/or COVID-19 was proposed [64].

At another Indian institution, cautioning regarding general, in-hospital SARS-CoV-2 acquisition risks was integrated into standard counselling for the obtainment of informed consent pre-operatively [43].

To achieve tumour shrinkage, avert local invasion and prevent complications until elective surgery could be safely performed–ideally *after* regional COVID-19 upsurges– 9 studies reported increased reliance on interim NACRT [7, 21, 23, 46, 58, 60, 64, 65, 67].

To elicit spontaneous colorectal cancer regression with non-operative treatment, clinicians at an Italian institution adopted a ‘watch-and-wait’ approach; however, this was acknowledged as controversial [23].

To prevent intraoperative SARS-CoV-2 aerosolisation, minimally invasive surgical techniques have been favoured [45, 58]. At a U.K. institution, robotic surgery was performed with the advantages of a shortened hospital stay and a decreased requirement for healthcare personnel intraoperatively [22]. However, several disadvantages were acknowledged, such as a need for dedicated OTs with appropriately-trained staff; that surgeons would be compelled to compromise on PPE utilisation whilst handling robotic equipment; a prolonged operative time requirement and a pre-requisite for extensive specialist input and increased nursing capacity [22].

To shorten hospital stay and optimise hospital bed capacity, 6 studies reported that patient discharges from hospital were expedited [21, 38, 61–63, 67].

Globally, non-urgent clinical activities (e.g., elective clinic appointments) were generally scaled back [55].

#### Infection prevention and control

Diligent hand hygiene and staff- and patient utilisation of PPE were widely emphasised [6, 18, 21, 24, 38, 58, 59, 61, 62, 66].

***Outcomes*** (S13 and S14 Tables)

### Metrics of quality

#### SARS-CoV-2 infection among healthcare personnel

Two Italian articles, a U.S. study and a French study reported on postoperative SARS-CoV-2 infection rates among healthcare personnel as an indicator of institutional response efficacy [6, 7, 38, 61]. In one study, this was measured by means of 4 nasopharyngeal SARS-CoV-2 tests per staff member; all (number unspecified) were uninfected [6].

#### Perioperative SARS-CoV-2 infection among patients

Among 11 studies, peri-operative SARS-CoV-2 infection rates among patients were measured as an indicator of health system response efficacy [5, 7, 18, 22, 26, 38, 40, 45, 49, 59, 63]. A Brazilian institution detected SARS-CoV-2 infection pre-operatively among n = 3 (4.41%) of 68 patients with breast cancer; this prompted a postponement of surgery by 21 days for each affected patient, to allow for recovery and repeat, post-convalescence reverse transcriptase polymerase chain reaction (RT-PCR) SARS-CoV-2 testing [18]. At another Brazilian centre, 41 (7.6%) infections among 540 patients were detected pre-operatively; of all affected patients, 5 (12.2%) had a diagnosis of breast cancer [5].

A global study, conducted across 55 countries, revealed that institutional “COVID-19-free surgical pathways” were associated with lower postoperative SARS-CoV-2 infection incidence (i.e., n = 53 [2.1%] of N = 2,481 patients operated on within ‘COVID-19-free’ pathways versus n = 238 [3.6%] of N = 6,820 patients treated within “no defined pathway”; adjusted odds ratio [aOR] 0.53 [95% CI, 0.36–0.76]) [59].

At a U.S. center, n = 1 of 92 patients from 5 (unspecified) surgical specialties tested positive for SARS-CoV-2, 18 days after elective cancer surgery [63]. A U.K.-based study found “no instances of in-patient coronavirus transmission[,]” among 60 patients who underwent elective robotic surgery for (unspecified) colorectal diagnoses (n = 10) [22].

#### Number of procedures performed without delay

Seven studies reported the number of elective procedures performed without delay and specified the case volume delayed due to pre-operative SARS-CoV-2 infection or, other pandemic- or patient-specific reasons [5, 7, 18, 22, 24, 26, 59, 60, 62, 66]. At an Italian centre, the median interval from primary colorectal cancer diagnosis to hospital admission for elective surgery was 23.1 days (range: 1–55 days) [21].

#### Postoperative complications, hospital stay, readmission, mortality

An international, multi-centre cohort study found that post-operative pulmonary complications (i.e., pneumonia, unexpected postoperative ventilation or acute respiratory distress syndrome) occur in approximately 50% of patients with peri-operative SARS-CoV-2 infection and are “associated with high mortality” (Table 6) [9]. Accordingly, 8 studies documented an observed absence of postoperative complications among their study cohorts, as potential evidence of health system response efficacy [7, 21, 24, 59, 61–63, 66].

**Table 6:** Mortality and pulmonary complications among patients with peri-operative SARS-CoV-2 infection.

No.	Authors (Year of publication)	Study design	Country	Diagnoses	Number of patients	Number of confirmed* pre-operative SARS-CoV-2 infections	30-day mortality	Incidence of pulmonary complications**
1.	Nepogodiev et al (2020a)	Cohort study	International (24 countries, 235 hospitals)	Any (i.e., not specific for breast- or colorectal cancer)	Total = 1,128 n = 280 (Elective surgery) n = 835 (Emergency surgery)	n = 294	n = 268 (23.8%)	n = 577 (51.2%)

*Pre-operative SARS-CoV-2 infection confirmed by: Laboratory-, radiologic or clinical diagnosis

** Pulmonary complications defined as: Pneumonia, Acute Respiratory Distress Syndrome (ARDS) or unexpected postoperative ventilation

At a U.K.-based institution, for robotic rectal cancer procedures within ‘COVID-19-protected’ units, a shortened median hospital stay of 4 days was documented–i.e., 2 days less than that observed pre-COVID-19, suggesting faster recovery [22]. This was partly attributed to higher healthcare provider-to-patient ratios [22]. At an Italian centre, the mean in-hospital stay was 2.2 days (standard deviation = 0.7 days) and no readmission was noted [26]. Another Italian study reported a median hospitalisation of 7.8 days (range: 4–18 days) [21]. Two further studies reported no readmission [5, 61].

In a U.S. setting, among 30 patients who underwent elective breast cancer surgery, over 90% demonstrated same-day discharge [38].

Within a U.K.-based COVID-19-protected surgical treatment pathway, of 168 patients who underwent colorectal cancer surgery (between April–June 2020), the 30-day mortality rate was 0.6% [41]. No COVID-19-related complications arose postoperatively or for 28 days post-discharge [41]. The readmission rate within 30 days post-discharge was 1.8% [41].

A U.S. single-center study documented 0% mortality among patients with breast- (n = 10), colon- (n = 14) or rectal (n = 2) cancer, one year post oncologic surgery, performed in September 2020 [42]. Internationally, 30-day postoperative mortality has been alluded to as an indicator of health system response efficacy, although this parameter has commonly been reported in a manner that reflects *all-cause* mortality; not necessarily mortality attributable to procedure deferrals during COVID-19 and/or to COVID-19 as a condition [56].

#### Tumour upstaging or regression

During the initial peak of worldwide COVID-19 disruption, delays to elective rectal cancer surgery lasting >62 days among 6 patients were reported in a U.K. setting; however, no histologic tumour upstaging was detected pre- or postoperatively [22]. Another U.K. study also described “[h]istopathologic outcomes…similar to normal practice[,]” suggesting no tumour upstaging secondary to delayed surgery [66]. However, ‘normal practice’ histopathology was not defined [66].

In another U.K. study, 22 of 49 patients with colorectal cancer were directed “straight to [elective] surgery” without prior neoadjuvant therapy; 9 of this sub-group (n = 22) displayed tumour progression, as compared to 3 of 27 (*p* = 0.0158) patients who did receive pre-operative, neoadjuvant therapy and displayed tumour advancement [40]. Of the 27 patients who received pre-operative neoadjuvant therapy, 7 displayed tumour regression [40].

## Discussion

In this scoping review of the global impact of the COVID-19 pandemic, we found insufficient evidence to support the preliminary hypothesis that greater mortality and vast surgical backlogs would be reported. However, institutional reductions in surgical activity compared to pre-pandemic periods were commonly observed, irrespective of the World Bank income stratification of the country-of-origin. These real-time estimates reflect the cumulative scale of global surgical backlogs and, by implication, the extent of urgent mitigation responses. The true, absolute magnitude of global backlogs remains unknown, partly due to the evolving nature of the pandemic and surgical service delivery. We identified a range of health system responses, mostly centered on COVID-19 mitigation and surgical case prioritisation, with some documentation of health system response efficacy (e.g., low hospital readmission rates) [26]. Most health system responses were universally applicable (e.g., hospital visitation restrictions), with some exceptions [63]. Most of the literature originated from HICs. Limited information was available on the context-specific impact and health system responses, implemented within LMICs. The majority of studies appraised were observational.

The existing evidence base suggests that elective surgery suspensions are not the sole cause of procedure backlogs [5]. Several intersecting, biopsychosocial and health system determinants exist [77]. For instance, pandemic-attributable suspensions of screening- and diagnostic investigations have also prolonged the time-to-surgery, following diagnosis [78, 79]. Thus, future breast and colorectal cases may be identified at later disease stages, resulting in a greater number of patients awaiting (higher-priority) surgery and/or increased mortality [80]. Individuals with breast- or colorectal cancer, by virtue of their clinical predisposition, may die from COVID-19 or other conditions, as opposed to prolonged time-to-surgery [9]. Comparing, quantifying and defining the cause of mortality among patients affected by procedure delays may thus pose a future methodological challenge. In 2013, an epidemiologic study, using data from the Korean Central Cancer Registry, demonstrated that for colorectal- and female breast cancer, the adjusted HRs for all-cause mortality, comparing a surgical delay lasting >12 weeks to the performance of surgery within 1–4 weeks of diagnosis were 2.65 (95% CI, 1.5–4.7) and 1.91 (95% CI, 1.06–3.49), respectively; no pattern of increased risk was noted for delays spanning 4–12 weeks [77]. Comorbidities, advanced-stage disease and high-income status, respectively, were associated with a faster time-to-surgery [77]. Whilst this evidence was published pre-COVID-19, it is useful to inform initial hypotheses of increased mortality that may arise secondary to extended, pandemic-attributable delays in elective breast- and colorectal cancer surgery [77].

Applying the social determinants of health (SDOH) framework to LMICs, long-term, mortality, backlogs, progression to inoperability and susceptibility to COVID-19 may be heightened, partly due to lower regional vaccination rates and/or finite health system capacity [81, 82]. Indeed, the epidemiologic study from Korea described income status and residential proximity to referral facilities as the most significant (social) determinants of delayed elective colorectal and female breast cancer surgery [77]. A limitation of most studies was that SDOH were not discussed as potential confounding or moderating factors. For instance, individual patients’ access to health insurance may have influenced institutional surgical activity [5]. Unless accounted for, such factors may hinder objective, inter-study comparisons of surgical activity. Timing is also a key consideration. Patient cohorts studied during a given timeframe may have been systematically different to other cohorts, sampled at alternative periods during regional SARS-CoV-2 infection upsurges (e.g., in terms of tendency to care-uptake hesitancy and ensuing voluntary surgery cancellation); hence, selection bias is possible [59, 83]. Further, healthcare personnel accounted for 14% of the global SARS-CoV-2 infection incidence, documented in September 2020 [84]. Thus, potential confounders, such as COVID-19, burn-out and absenteeism among healthcare personnel, may obscure the extent to which any difference in pre-pandemic versus current surgical backlogs may be ascribed to chance [85].

COVID-19 was declared a global pandemic on 11 March 2020 [86]. Many of the included studies utilised data from the initial 12–16 weeks of the pandemic (i.e., March–May 2020). This implies that the data were collected contemporaneously during peak pandemic disruption, worldwide. This methodologic strength may have minimised recall bias among respondents and researchers. Further, among the cohort studies, specifically, temporality between exposures (e.g., surgery within a ‘COVID-19-protected’ pathway) and outcomes (e.g., postoperative SARS-CoV-2 infection) could be inferred [59].

This review has some limitations. The institutional study populations were small, conferring limited statistical power and constrained generalisability of the findings to greater regional or global populations. There was limited evidence available on clinical outcomes among patients (notably, mortality). The wider literature on other cancers was excluded from our review on breast and colorectal cancer. Only English-language publications were included; this may have undermined the completeness of the evidence base appraised.

An explanation for the paucity of evidence on mortality may be the relatively short, (insufficient) period that has elapsed since December 2019, considering that cancer tends to advance gradually. To detect appreciable differences in mortality outcomes, a longer, feasible follow-up timeframe for possible disease progression to inoperability, life-threatening complications or mortality would be required. At this juncture, significant differences in mortality may be less likely, assuming that ostensibly, the majority of deprioritised patients would have earlier-stage disease. An alternative explanation may be the effect of publication bias; studies that revealed no significant difference in measured mortality outcomes may not have been published. Additionally, “[f]ew countries have [had] access to real-time data,” and there may be delays in the publication of new evidence due to pandemic-associated “pressures on health systems[,]” worldwide [27].

Regarding procedure backlogs, the concern of lead-time bias arises. ‘Lead-time’ denotes the time between the initial disease detection and a measured outcome. A comparison of surgical activity across studies may be inappropriate because patient subgroups with early-stage disease may have been deprioritised for surgical intervention; the data for such subgroups may consequently reflect lower institutional surgical activity [7]. The concern of lead-time bias is applicable also to other outcomes, such as mortality, pre-operative SARS-CoV-2 infection, postoperative complications, hospital readmission and prolonged hospital stay–i.e., patient cohorts with early-stage disease may be less likely to demonstrate these outcomes [87]. Accounting for patient- (e.g., age, sex, comorbidities) and tumour-specific (e.g., Human epidermal growth factor receptor-2 [HER-2]-positivity) factors would also be essential to counter effect modification in mortality, surgical activity- or procedure backlog outcomes. Ostensibly, such characteristics would influence clinical decision-making regarding patients’ individual risk profiles and their prioritisation for earlier surgical intervention [21]. The American Cancer Society (2019) states that HER-2-receptor-positive breast tumours are commonly treated with endocrine (non-surgical) therapy; hence, patients with this tumour characteristic may be predisposed to delayed elective surgery, in favour of interim, non-operative treatment instead [88]. Most studies reported crude surgical case performance rates and did not adjust, stratify or conduct sensitivity analysis, according to the clinical factors and potential confounders, outlined above.

Some health system responses implemented within HICs (e.g., robotic surgery), may have limited generalisability to more resource-limited settings within LMICs [22]. Observer-expectancy bias may have influenced reporting on the efficacy of some health system responses, such as ‘COVID-19-protected’ pathways, because none of the studies instituted blinding for researchers or participants.

Regarding health system response efficacy, there is potential for misclassification bias in the comparison and quantification of differences in outcomes. This applies in instances where heterogeneity exists in the definitions and metrics utilised across studies (e.g., in some studies, pre-operative SARS-CoV-2 infection was defined by a positive symptom screen, whereas others relied on objective laboratory testing or thoracic CT to ascertain SARS-CoV-2 infection status) [7]. The same principle applied to the diagnosis of breast- or colorectal cancer–i.e., some studies may have relied on clinical assessments; whereas, others may have utilised laboratory markers or radiologic findings or, a combination of all three indices for defining diagnoses [9]. Studies that involved COVID-19 symptom screens may have been subject to information bias, as SARS-CoV-2 infection may have remained undetected among asymptomatic patients and/or patients’ subjective self-reporting may have been influenced by social desirability (e.g., a desire to access expedited surgery) [26]. Patients’ demographic characteristics (e.g., age) and clinical risk profiles (e.g., prior treatment exposures) may have also confounded the association between health system responses and the incidence of postoperative complications, readmission rates, length of hospital stay and SARS-CoV-2 infection incidence, respectively [89]. Reliance on these parameters as indicators of health system response efficacy is problematic, as patients with cancer are clinically predisposed to these adverse outcomes; potential selection bias is thus apparent [90]. Furthermore, because many patients’ discharge (to home-based care) was expedited, it may be difficult to exclude post-operative community acquisition of SARS-CoV-2 [62]. Therefore, this parameter may be less valid as a health system response quality indicator. Other patient- or pandemic-specific factors, such as tobacco use, treatment non-adherence and decreased follow-up during lockdowns, for example, may have obfuscated the true efficacy of health system responses [12]. Lastly, deducing health system response efficacy from SARS-CoV-2 infection incidence among healthcare personnel may introduce selection bias, as this population subgroup may be more inclined to adhere to COVID-19 prevention measures [91].

Collectively, these sources of potential bias, chance, effect modification and confounding generate a risk of over- or understating the impact of COVID-19.

## Conclusions

Breast- and colorectal cancer contribute significantly to the global burden of disease. With early detection and timely treatment, the conditions may be curable. Global suspensions of elective, oncologic surgery during the COVID-19 pandemic carry adverse ethical, quality-of-care- and economic implications. Unfortunately, evidence on global case performance backlogs remains scarce. Additionally, there is currently no evidence available to demonstrate that elective breast- and colorectal cancer surgery deferrals during the COVID-19 global pandemic led to increased patient mortality. None of the reviewed primary research studies reported on mortality attributable to delayed surgical intervention during COVID-19. Whilst this is partly due to the limited time that has elapsed since SARS-CoV-2 emerged in December 2019, the lack of robust, real-time surveillance of cancer outcomes has exacerbated this evidence gap. Based on previously conducted systematic reviews, we may surmise that delays in surgical intervention may lead to poorer clinical outcomes, including higher mortality. Reassuringly, to maintain service delivery for high-risk patients and simultaneously mitigate contagion, various health system responses have been implemented worldwide, many of which are universally applicable. In the absence of robust global measures of implemented health system responses and cancer outcomes, it is challenging to evaluate the efficacy of the former in mitigating health service disruption. This is particularly applicable to LMICs. Future research should focus on strategies to improve global, cancer-related outcome surveillance, and the measurement of health system responses and their efficacy. Clinical and global health practice is evolving iteratively in response to the pandemic and its widespread population health- and socioeconomic effects. A future systematic review on this topic is recommended once further evidence is available.

## Supporting information

S1 Text Definitions. (DOCX)

S2 Text Research statement. (DOCX)

S1 Data List of data items populated in data extraction form. (DOCX)

S1 Checklist Preferred Reporting Items for Systematic Reviews and Meta-analyses extension for Scoping Reviews (PRISMA-ScR) checklist. (DOCX)

S1 Table Population, Exposure, Comparator, Outcome (PECO) components. (DOCX)

S2 Table Primary domains of search strategy. (DOCX)

S3 Table Search strategy executed via *Ovid* interface on 24 November 2022. (DOCX)

S4 Table Overall survival outcomes for delayed elective colorectal cancer surgery during the COVID-19 pandemic. (DOCX)

S5 Table Structural health system responses for elective breast surgery delays. (DOCX)

S6 Table Further structural health system responses for elective breast- or colorectal cancer surgery. (DOCX)

S7 Table Health policy responses for elective breast cancer surgery delays. (DOCX)

S8 Table Health policy responses for elective breast- and colorectal cancer surgery delays. (DOCX)

S9 Table Human resources responses for elective breast cancer surgery delays. (DOCX)

S10 Table Human resources responses for elective colorectal cancer surgery delays. (DOCX)

S11 Table Adapted healthcare provision processes for elective breast cancer surgery delays. (DOCX)

S12 Table Adapted healthcare provision processes for elective breast- and colorectal cancer surgery delays. (DOCX)

S13 Table Clinical outcomes as health system response efficacy indicators for delays in elective breast cancer surgery. (DOCX)

S14 Table Clinical outcomes as health system response efficacy indicators for delays in elective breast- and colorectal cancer surgery. (DOCX)

## Abbreviations

CASP: Critical Appraisal Skills Programme
CI: Confidence Interval
COVID-19: Coronavirus disease-2019
CRC: Colorectal cancer
DALY: Disability-adjusted life year
HCP: Healthcare personne
HER-2 receptor: Human epidermal growth factor receptor-2
HIC: High-income country
HSR: Health system response
LMIC: Low- or middle-income country
MeSH: Medical subject heading
NACRT: Neoadjuvant chemoradiotherapy
No: Number
OT: Operating theatre
PPE: Personal Protective Equipment
PRISMA: Preferred Reporting Items for Systematic Reviews and Meta-Analyses
SARS-CoV-2: Severe Acute Respiratory Syndrome Coronavirus-2
SDI: Socio-demographic index
U.K: United Kingdom
U.S: United States
UMIC: Upper middle-income country
WHO: World Health Organization
