# Correction: Global elective breast- and colorectal cancer surgery performance backlogs, attributable mortality and implemented health system responses during the COVID-19 pandemic: A scoping review

**DOI:** 10.1371/journal.pgph.0002364

**Published:** 2023-09-01

**Authors:** The PLOS Global Public Health Staff

There are errors in the Funding statement. The publisher apologizes for the errors. The correct Funding statement is as follows: SH received a scholarship from Africa Health Research Institute and University College London, for her postgraduate programme of study at University College London (in 2020–2021), during which this study was undertaken. MS is an NIHR Research Professor (NIHR 301634). MS also receives funding from the U.S. National Institute of Health (NIH) R01 (5R01MH114560-03); Wellcome Trust (082384/Z/07/Z) and Bill and Melinda Gates Foundation (INV-0336500). Publication fees for this review were funded by the Wellcome Trust (Grant number for Wellcome Trust Strategic Core award: 201433/Z/16/A). For the purpose of open access, the authors have applied a CC BY public copyright licence to any Author Accepted Manuscript version arising from this submission. The funders had no role in the study design, data collection and analysis, decision to publish, or preparation of the manuscript.
