# Supplementary material for: Global elective breast- and colorectal cancer surgery performance backlogs, attributable mortality and implemented health system responses during the COVID-19 pandemic: A scoping review
Source: PLOS Glob Public Health. 2023 Apr 4;3(4):e0001413. doi: 10.1371/journal.pgph.0001413 (PMC10072489; doi:10.1371/journal.pgph.0001413)
Supplement: S1 Text — (DOCX) [file pgph.0001413.s001.docx]

**S1 Text: Definitions**

1. **Capsule colonoscopy** – A procedure whereby a patient swallows a small, pill-sized capsule device that traverses the digestive tract, including the colon and rectum. The capsule consists of a light source and camera, which enable visualisation and (external) computer imaging of the internal anatomy of the digestive tract. The procedure is considered less invasive to standard colonoscopy, which necessitates the insertion of an endoscope into the digestive tract for visualisation of the internal anatomy [1]
2. **‘COVID-19-protected’ hospital pathway** – Also known as ‘COVID-19-free surgical pathway’ or ‘COVID-19 green pathway’. During the COVID-19 pandemic, to prevent outbreaks of hospital-acquired SARS-CoV-2 infection, hospital ‘pathways’ (i.e., structures and protocols) were re-organised and adapted. The aim was to minimise the exposure of patients with cancer to other patients, personnel, individuals, hospital units or objects, which could be potential sources of SARS-CoV-2 transmission. Generally, the revised hospital pathways combined and operationalised structural measures (e.g., physical distancing of hospital beds) with adapted healthcare provision practices (e.g., use of facemasks during consultations) for COVID-19 mitigation [2].
3. **Elective surgery** – Any procedure scheduled in advance, with local, regional or general anaesthesia, where delays in such an intervention would not cause imminent mortality or severe morbidity [3, 4]
4. **Emergent surgery** – Any procedure prioritised for immediate or urgent performance, with local, regional or general anaesthesia, where delays in such an intervention would likely cause imminent mortality or severe morbidity [3, 4]
5. **Healthcare personnel (HCP)** – Denoting, in this review, health facility staff members who are directly involved in the provision of healthcare to patients; examples include nurses, paramedics, surgeons, anaesthetists, other clinicians, allied health professionals, respiratory therapists, pharmacists, medical and nursing students and/or medical volunteers [5]
6. **Socio-demographic index** – Summary measure (ranging from 0 to 1) of individual countries’ development stratification; the measure combines ranking of the per capita income, the average educational attainment and regional fertility rates, as included in global burden of disease estimates [6]
7. **Metastasis** – Cancer dissemination from the primary site to other organ systems; metastasis constitutes the most advanced stage of cancer and is generally incurable. It is usually treated with supportive, palliative care [7]
8. **Benign** – Lacking potential for metastasis [7]
9. **Malignant** – Having potential for metastasis [7]
10. **Staging** – Determination of the extent of cancer present in the body, to guide treatment and estimate the prognosis; cancer stages generally range from 0 to IV. Lower stages indicate more localised disease and higher stages signify an adversely more advanced extent of cancer dissemination [8, 9]
11. **Grading** – Extent to which aberrant tumour cells resemble normal cells; the higher the grade, the lower the resemblance and thus, the more aggressive the disease and, vice versa [8]
12. **Histology, histopathology** – Microscopic study of tissue specimens, including the analysis of changes caused by a disease; typically this analysis is undertaken for grading and staging purposes [10]
13. **Laparoscopic resection** – Surgery performed with a laparoscope (i.e., thin, cylindrical instrument with a lens and light source), for removal of tissue [11]
14. **Thoracic computed tomography (CT)** – Radiologic imaging scan of the chest, including the lungs [12]
15. **Reverse transcriptase polymerase chain reaction (RT-PCR)** – Diagnostic laboratory test that may be used for the detection of SARS-CoV-2 viral genetic material (e.g., on a throat swab specimen) [13]

**References:**

1. U.S. National Cancer Institute. Capsule endoscope [Internet]. 2022. [Acessed 8 November 2022]. Available from: <https://www.cancer.gov/publications/dictionaries/cancer-terms/def/capsule-endoscope>
2. Boffa DJ, Judson BL, Billingsley KG, Del Rossi E, Hindinger K, Walters S, et al. Results of COVID-minimal surgical pathway during surge-phase of COVID-19 pandemic. Ann Surg [Internet]. 2020. [Accessed 2022 Aug 28]; 272(6):e316-e20. Available from: <https://doi.org/10.1097%2FSLA.0000000000004455>
3. Shlobin NA, Rosenow JM, Ford PJ. Using functionality rather than elective nature to characterize neurosurgeries during pandemic triage. Am J Bioeth [Internet]. 2020. [Accessed 2021 Aug 29]; 20(7): 196-8. Available from: <https://doi.org/10.1080/15265161.2020.1777353>
4. Nepogodiev D., Omar O.M., Glasbey J.C., et al. 2020b. Elective surgery cancellations due to the COVID-19 pandemic: Global predictive modelling to inform surgical recovery plans. Br J Surg [Internet]. 2020. [Accessed 2021 Aug 29]; 107(11): 1440-9. Available from: <https://doi.org/10.1002/bjs.11746>
5. U.S. Centers for Disease Control and Prevention (CDC). Recommended vaccines for healthcare workers [Internet]. 2 May 2016. [Accessed 29 August 2021]. Available from: <https://www.cdc.gov/vaccines/adults/rec-vac/hcw.html>
6. University of Washington Institute for Health Metrics and Evaluation. Socio- demographic index [Internet]. 2020. [Accessed 29 August 2021]. Available from: <http://www.healthdata.org/taxonomy/glossary/socio-demographic-index-sdi>
7. World Health Organization (WHO). Cancer [Internet]. 3 February 2022. [Accessed 25 January 2023]. Available from: <https://www.who.int/news-room/fact-sheets/detail/cancer>
8. American Cancer Society. Cancer staging [Internet]. 2022. [Accessed 2 August 2022]. Available from: <https://www.cancer.org/treatment/understanding-your-diagnosis/staging.html>
9. U.S. National Cancer Institute (UNCI). Cancer staging [Internet]. 9 March 2015. [Accessed 29 August 2021]. Available from: <https://www.cancer.gov/about-cancer/diagnosis-staging/staging>
10. U.S. National Cancer Institute. Histology [Internet]. 2021. [Accessed 29 August 2021]. Available from: <https://www.cancer.gov/publications/dictionaries/cancer-terms/def/histology>
11. U.S. National Cancer Institute. Laparoscopic-assisted resection [Internet]. 2021. [Accessed 29 August 2021]. Available from: <https://www.cancer.gov/publications/dictionaries/cancer-terms/def/laparoscopic-assisted-resection>
12. Huddy JR, Crockett M, Nizar AS, Smith R, Malki M, Barber N, et al. Experiences of a "COVID protected" robotic surgical centre for colorectal and urological cancer in the COVID-19 pandemic. J Robot Surg [Internet]. 2021. [Accessed 2021 Aug 29]; 16(1):59-64. Available from: <https://doi.org/10.1007/s11701-021-01199-3>
13. Di Marzo F, Fiori E, Sartelli M, Cennamo R, Coccolini F, Catena F, et al. SARS-CoV-2 pandemic: Implications in the management of patients with colorectal cancer. New Microbiol [Internet]. 2020. [Accessed 2021 Aug 29]; 43(4):156-60. Available from: [https://pubmed.ncbi.nlm.nih.gov/330213 20/](https://pubmed.ncbi.nlm.nih.gov/33021320/)
