# Supplementary material for: Global elective breast- and colorectal cancer surgery performance backlogs, attributable mortality and implemented health system responses during the COVID-19 pandemic: A scoping review
Source: PLOS Glob Public Health. 2023 Apr 4;3(4):e0001413. doi: 10.1371/journal.pgph.0001413 (PMC10072489; doi:10.1371/journal.pgph.0001413)
Supplement: S2 Text — (DOCX) [file pgph.0001413.s002.docx]

**S2 Text:**

**Literature review: Preliminary research statement (2021)**

**The impact of the COVID-19 pandemic on the global delivery of non-emergency cancer surgery and, potential strategies to address this challenge**

Sonia Haribhai^1,2*^, Komal Bhatia^1^ and Maryam Shahmanesh^1,2^

1: Institute for Global Health, University College London, London, United Kingdom

2: Africa Health Research Institute, Durban, South Africa

**Corresponding author:** Sonia Haribhai (*sonia.haribhai@gmail.com)

________________________________________________________________________________

**Background**

The coronavirus disease-2019 (COVID-19) global pandemic has placed unprecedented strain on lives, health systems and economies, worldwide [1]. Thus far, over 132 million confirmed cases have been recorded, with over 2.8 million fatalities [2]. In the interests of infection prevention and control and, the redirection of healthcare resources and personnel to the COVID-19 response, healthcare facilities within many high-income- and low-and-middle-income countries (LMICs) suspended non-emergency surgery services [3, 4]. Consequently, significant backlogs of non-emergency surgical cases, including oncological procedures, have accrued and, affected patients have faced uncertainty, persistent ailments and incomplete care [5]. Increased health workforce capacity and the formulation of evidence-based guidelines have been identified as strategies to facilitate the urgent, ethical and equitable prioritisation of elective surgery services during the pandemic [3].

This project will describe the impact of the COVID-19 pandemic on the global delivery of non-emergency cancer surgery and identify potential strategies to address this challenge. The impact will be defined by the outcomes summarised below.

**PECO framework [6] – Provisional summary**

- **Population**:
  - Adults (>18 years) requiring non-emergency cancer surgery within any country
- **Exposure**:
  - Non-emergency cancer surgery, deferred due to the COVID-19 pandemic
- **Comparator**:

- Current vs. pre-pandemic backlogs, within particular settings

- Current vs. pre-pandemic outcomes among patients affected by delays, within particular settings

- **Outcomes**:
  - Morbidity, mortality, inoperability and surgical backlogs

**Methods**

The project will consist of a scoping review of articles published from December 2019 to the present date. Articles based on pre-pandemic outcomes, published within the past 10 years, will be reviewed for comparison. We will search the Medline (using the *PubMed* interface) and Embase databases, as these encompass biomedicine articles from international sources. We aim to apply Preferred Reporting Items for Systematic Reviews and Meta-analyses (PRISMA) guidelines [7] and develop a replicable search strategy, prioritising the inclusion of peer-reviewed, academic journal articles. The literature search will consist of a combination of three primary domains as Medical Subject Headings (MeSH) with keywords (Table 1). The results from quantitative studies will be combined with key points from qualitative studies and review articles, in a narrative synthesis. We will present the results thematically to yield an overall synopsis of the issue.

**Table 1 – Search strategy: Primary domains, search terms & synonyms**

| **No.:** | **Primary domain search terms:** | **Keyword examples:** |
| --- | --- | --- |
| 1. | COVID-19 | SARS-CoV-2  Novel coronavirus  COVID-19 |
| 2. | Elective surgery | Non-emergency surgery  Elective procedure*  Elective surgical procedure* |
| 3. | Cancer  (Specific cancer types that will be the focus areas are still to be confirmed) | Malignan*  Tumour  Neoplasm |

**Perceived challenges**

Anticipated challenges include a scarcity of literature on the context-specific impact and responses within LMICs. Additionally, we are still narrowing down the type of cancer surgery (e.g., prostate cancer surgery) that will be our focus. It will pose a methodological challenge to compare and quantify the difference in outcomes, attributable to the COVID-19 pandemic. Individuals with cancer represent a clinically vulnerable population subgroup; many may have died from COVID-19, as opposed to delays in surgical intervention. There is a risk of over- or understating the impact. We aim to keep up-to-date with (contemporaneously emerging) publications and will declare any major challenges encountered, as limitations of the final review.

**Motivation**

We are interested to investigate how surgical disciplines, targeted at patients with cancer, have prepared for resilience against COVID-19, as a global health crisis, through both upstream (e.g., national frameworks) and downstream (e.g., health service outsourcing) interventions.

**References**

1. World Health Organization (WHO). Urgent health challenges for the next decade [Internet]. 13 January 2020. [Accessed 9 April 2021]; Available from: <https://www.who.int/news-room/photo-story/photo-story-detail/urgent-health-challenges-for-the-next-decade>
2. World Health Organization (WHO). WHO Coronavirus Disease (COVID-19) Dashboard [Internet]. 2021. [Accessed 9 April 2021]; Available from: https://covid19.who.int
3. Al-Omar, K., Bakkar S., Khasawneh L., Donatini G. and Miccoli P. Resuming elective surgery in the time of COVID-19: A safe and comprehensive strategy. Updates in Surgery [Internet]. 2020. [Accessed 2021 April 11]; 72(2), p.291-295. Available from: <https://doi.org/10.1007/s13304-020-00822-6>
4. Chu KM, Owolabi EO, Smith M, Hardcastle TC, Maswime S, Geduld H, et al. Establishing a South African national framework for COVID-19 surgical prioritisation. South African Medical Journal [Internet]. 2021. [Accessed 2021 April 11]. Available from: <http://www.samj.org.za/index.php/samj/article/view/13237/9712>
5. Anon. Too long to wait: The impact of COVID-19 on elective surgery. The Lancet Rheumatology [Internet]. 2021. [Accessed 2021 April 11]; 3(2), e83. Available from: <https://doi.org/10.1016/S2665-9913(21)00001-1>
6. Morgan RL, Whaley P, Thayer KA and Schunemann HJ. Identifying the PECO: A framework for formulating good questions to explore the association of environmental and other exposures with health outcomes. Environ Int. 2018. [Accessed 2023 March 14]; 121(Pt 1):1027-31. Available from: <https://doi.org/10.1016%2Fj.envint.2018.07.015>
7. Tricco AC, Lillie E, Zarin W, O’Brien KK, Colquhoun H, Levac D, et al. PRISMA Extension for Scoping Reviews (PRISMA-ScR): Checklist and explanation. Ann Intern Med [Internet]. 2018. [Accessed 2023 Mar 14]; 169(7): 467-473. Available from: <https://knowledgetranslation.net/portfolios/the-prisma-scr-prisma-extension-for-scoping-reviews/>
