# Supplementary material for: Global elective breast- and colorectal cancer surgery performance backlogs, attributable mortality and implemented health system responses during the COVID-19 pandemic: A scoping review
Source: PLOS Glob Public Health. 2023 Apr 4;3(4):e0001413. doi: 10.1371/journal.pgph.0001413 (PMC10072489; doi:10.1371/journal.pgph.0001413)
Supplement: S1 Data — (DOCX) [file pgph.0001413.s003.docx]

**S1 Data** – Data item headings for data extraction form

(Note: Some data items were optional for entry and/or entered separately.)

- Database (MEDLINE/EMBASE)
- Date of database search: 24 November 2022
- Heading 1: MEDLINE BREAST CANCER ARTICLES
- Heading 2: EMBASE BREAST CANCER ARTICLES
- Heading 3: MEDLINE COLORECTAL CANCER ARTICLES
- Heading 4: EMBASE COLORECTAL CANCER ARTICLES
- Heading 5: EMBASE BREAST AND COLORECTAL CANCER ARTICLES
- (0 search results available for EMBASE BREAST AND COLORECTAL CANCER MeSH terms combined)
- Author
- Date of publication
- Country-of-origin
- Study design
- Journal
- Language
- Cancer type (Breast/Colorectal/Both)
- Pre-print: (Yes/No)
- Include/Exclude/Maybe
- Rationale (Aim of study)
- Peer-reviewed (Yes/No)
- Grey literature (Yes/No)
- Link to article (online)
- Grouping: (IMPACT: Backlogs or mortality/HSRs/BOTH)
- Number of patients needing elective surgery
- Number of cases performed
- Number of cases performed (pre-pandemic group) (if applicable)
- Case performance reduction (%)
- Study period (dates)
- Outcome determinants
- Patient characteristics recorded (e.g., age, sex, other)
- Description: Health system responses
- Outcome measures: Metrics of HSR efficacy (e.g., hospital readmission)
- Quality rating (Good/Intermediate/Poor)
- Reason for quality rating
- Comments: Bias
- Comments: Chance
- Comments: Confounding
- Additional notes

Number of articles for each designation – i.e.: ‘EMBASE breast cancer’; ‘MEDLINE breast cancer’; ‘EMBASE colorectal cancer’; ‘MEDLINE colorectal cancer’)
