# Supplementary material for: Global elective breast- and colorectal cancer surgery performance backlogs, attributable mortality and implemented health system responses during the COVID-19 pandemic: A scoping review
Source: PLOS Glob Public Health. 2023 Apr 4;3(4):e0001413. doi: 10.1371/journal.pgph.0001413 (PMC10072489; doi:10.1371/journal.pgph.0001413)
Supplement: S1 Table — (DOCX) [file pgph.0001413.s005.docx]

**S1 Table** – Population, Exposure, Comparator, Outcome (PECO) components

| **No.** | **Component:** | **Description:** |
| --- | --- | --- |
| 1. | Population | - Adults (age: ≥18 years) requiring elective breast- or colorectal cancer surgery, within any country |
| 2. | Exposure | - Elective breast- or colorectal cancer surgery, deferred due to the COVID-19 pandemic |
| 3. | Comparator | Global, regional, local or institutional:   - Current versus pre-pandemic number of procedures performed (i.e., surgical activity) - Number of procedures performed versus case volume of backlog |
| 4. | Outcome | - Elective surgery backlogs, attributable mortality and health system responses, implemented to address this challenge |

Adapted from –

Morgan RL, Whaley P, Thayer KA and Schunemann HJ. Identifying the PECO: A framework for formulating good questions to explore the association of environmental and other exposures with health outcomes. Environ Int. 2018. [Accessed 2023 March 14]; 121(Pt 1):1027-31. Available from: <https://doi.org/10.1016%2Fj.envint.2018.07.015>
