# Supplementary material for: Global elective breast- and colorectal cancer surgery performance backlogs, attributable mortality and implemented health system responses during the COVID-19 pandemic: A scoping review
Source: PLOS Glob Public Health. 2023 Apr 4;3(4):e0001413. doi: 10.1371/journal.pgph.0001413 (PMC10072489; doi:10.1371/journal.pgph.0001413)
Supplement: S2 Table — (DOCX) [file pgph.0001413.s006.docx]

**S2 Table**– Primary domains of search strategy

| **No.:** | **Domain:** | **Medical Subject Heading (MeSH) (MEDLINE):** | **Medical Subject Heading (MeSH) (EMBASE):** | **Some keywords used:** |
| --- | --- | --- | --- | --- |
| 1. | COVID-19  **AND** | COVID-19 | Coronavirus disease 2019 | “SARS-CoV-2”  “Novel coronavirus” |
| 2. | Elective surgery  **AND** | Elective surgical procedures | Elective surgery | “Non-emergen* surg*”  “Elective operation*” |
| 3. | Cancer  **AND** | Neoplasms | Malignant neoplasm | “Cancer*”  “Tumour*, tumor*” |
| 4. | Breast cancer  **AND** | Breast neoplasms | Breast cancer | “Breast cancer”  “Breast tumour*” |
| 5. | Colorectal cancer | Colorectal neoplasms | Colorectal cancer | “Colorect* cancer*”  “Colorect* tumour*”  “Colo* malignan*”  “Rect* neoplas*” |
| **Search domain combinations implemented in MEDLINE and EMBASE**: | | | | |
| A | 1 and 2 and 3 and 4 | | | |
| B | 1 and 2 and 3 and 5 | | | |
| C | 1 and 2 and 3 and 4 and 5 | | | |
| D | 1 and 2 and 3 | | | |
