# Supplementary material for: Global elective breast- and colorectal cancer surgery performance backlogs, attributable mortality and implemented health system responses during the COVID-19 pandemic: A scoping review
Source: PLOS Glob Public Health. 2023 Apr 4;3(4):e0001413. doi: 10.1371/journal.pgph.0001413 (PMC10072489; doi:10.1371/journal.pgph.0001413)
Supplement: S3 Table — (DOCX) [file pgph.0001413.s007.docx]

**S3 Table**– Search strategy executed via *Ovid* interface on 24 November 2022

**Note**: The date restriction of “2019-Current” may be interpreted as “2019 up to and including 24 November 2022” because 24 November 2022 was the date of the search execution and thus constitutes the upper date limit.

| **MEDLINE** | **EMBASE** |
| --- | --- |
| 1. exp COVID-19/ 2. Limit 1 to yr=“2019-Current” 3. (“SARS-CoV-2” or “Severe Acute Respiratory Syndrome Coronavirus 2” or “Severe Acute Respiratory Syndrome Coronavirus-2” or “SARS CoV 2” or “SARS CoV-2” or “COVID-19” or “COVID 19” or “coronavirus disease 2019” or “coronavirus disease-2019” or “coronavirus” or “novel coronavirus”) 4. Limit 3 to yr=“2019-Current” 5. 1 or 4 6. exp Neoplasms/ 7. Limit 6 to yr=“2019-Current” 8. (cancer* or tumor* or tumour* or “oncolog*” or “neoplasm” or “neoplas*” or “benign” or “malignan*” or “metastatic” or “metastases” or “metasta*”) 9. Limit 8 to yr=“2019-Current” 10. 7 or 8 11. exp Breast Neoplasms/ 12. Limit 11 to yr=“2019-Current” 13. (“breast cancer” or “breast neoplas*” or “breast tumor*” or “breast tumour*” or “breast mass” or “breast lump*” or “neoplas* breast” or “malignant brease disease” or “benign breast disease” or “breast oncolog*” or “breast carcinoma” or “ductal carcinoma in situ” or “invasive breast cancer” or “invasive breast carcinoma” or “invasive lobular carcinoma” or “invasive ductal carcinoma” or “triple negative breast cancer” or “inflammatory breast cancer” or “Paget disease of the breast” or “breast angiosarcoma” or “breast sarcoma” or “Phyllodes tumor*” or “Phyllodes tumour*”) 14. Limit 13 to yr=“2019-Current” 15. 12 or 14 16. exp Colorectal Neoplasms/ 17. Limit 16 to yr=“2019-Current” 18. (“colorectal cancer” or “colorectal carcinoma” or “colorectal tumor*” or “colorectal tumour*” or “colorectal mass*” or “colorectal oncolog*” or “colorectal neoplas*” or “neoplas* colorectal” or “colorectal malignan*” or “malignan* colorectal” or “benign colorectal” or “colorectal polyp*” or “colo* cancer” or “colo* carcinoma” or “colo* tumor” or “colo* tumour” or “colo* mass” or “colo* neoplas*” or “colo* polyp” or “colo* malignan*” or “colo* metasta*” or “metasta* colo*” or “colorectal adenocarcinoma” or “colo* adenocarcinoma” or “gastrointestinal carcinoid tumor*” or “gastrointestinal carcinoid tumour*” or “GIT carcinoid tumor*” or “GIT carcinoid tumour*” or “colo* lymphoma*” or “colo* sarcoma*” or “colo* adenoma*” or “colo* adenomatous polyp*” or “rect* cancer” or “rect* tumor*” or “rect* tumour*” or “rect* oncolog*” or “rect* carcinoma*” or “rect* mass*” or “rect* malignan*” or “neoplas* rect*” or “rect* neoplas*” or “anal cancer*” or “anal carcinoma*” or “anal mass*” or “anus cancer*” or “anus carcinoma*”) 19. Limit 18 to yr=“2019-Current” 20. 17 or 19 21. exp Elective Surgical Procedures/ 22. Limit 21 to yr=“2019-Current” 23. (“elective surgery” or “elective surgical procedure*” or “elective surg*” or “elective operation*” or “elective surg* intervention*” or “non-emergen* surgery” or “non-emergen* surg*” or “non-emergen* surgical procedure*” or “non-emergen* surg*” or “non-emergen* operation*” or “non-emergen* procedure*” or “elective procedure*” or “elective surg* intervention*” or “non-emergency surg* intervention*” or “non-emergen* operation*”) 24. Limit 23 to yr=“2019-Current” 25. 22 or 24 26. 5 and 10 and 25 27. 5 and 10 and 15 and 25 28. 5 and 10 and 20 and 25 29. 5 and 10 and 15 and 20 and 25 | 1. exp coronavirus disease 2019/ 2. Limit 1 to yr=“2019-Current” 3. (“SARS-CoV-2” or “Severe Acute Respiratory Syndrome Coronavirus 2” or “Severe Acute Respiratory Syndrome Coronavirus-2” or “SARS CoV 2” or “SARS CoV-2” or “COVID-19” or “COVID 19” or “coronavirus disease 2019” or “coronavirus disease-2019” or “coronavirus” or “novel coronavirus”) 4. Limit 3 to yr=“2019-Current” 5. 1 or 4 6. exp malignant neoplasm/ 7. Limit 6 to yr=“2019-Current” 8. (cancer* or tumor* or tumour* or “oncolog*” or “neoplasm” or “neoplas*” or “benign” or “malignan*” or “metastatic” or “metastases” or “metasta*”) 9. Limit 8 to yr=“2019-Current” 10. 7 or 8 11. exp breast cancer 12. Limit 11 to yr=“2019-Current” 13. (“breast cancer” or “breast neoplas*” or “breast tumor*” or “breast tumour*” or “breast mass” or “breast lump*” or “neoplas* breast” or “malignant breast disease” or “benign breast disease” or “breast oncolog*” or “breast carcinoma” or “ductal carcinoma in situ” or “invasive breast cancer” or “invasive breast carcinoma” or “invasive lobular carcinoma” or “invasive ductal carcinoma” or “triple negative breast cancer” or “inflammatory breast cancer” or “Paget disease of the breast” or “breast angiosarcoma” or “breast sarcoma” or “Phyllodes tumor*” or “Phyllodes tumour*”) 14. Limit 13 to yr=“2019-Current” 15. 12 or 14 16. exp colorectal cancer/ 17. Limit 16 to yr=“2019-Current” 18. (“colorectal cancer” or “colorectal carcinoma” or “colorectal tumor*” or “colorectal tumour*” or “colorectal mass*” or “colorectal oncolog*” or “colorectal neoplas*” or “neoplas* colorectal” or “colorectal malignan*” or “malignan* colorectal” or “benign colorectal” or “colorectal polyp*” or “colo* cancer” or “colo* carcinoma” or “colo* tumor” or “colo* tumour” or “colo* mass” or “colo* neoplas*” or “colo* polyp” or “colo* malignan*” or “colo* metasta*” or “metasta* colo*” or “colorectal adenocarcinoma” or “colo* adenocarcinoma” or “gastrointestinal carcinoid tumor*” or “gastrointestinal carcinoid tumour*” or “GIT carcinoid tumor*” or “GIT carcinoid tumour*” or “colo* lymphoma*” or “colo* sarcoma*” or “colo* adenoma*” or “colo* adenomatous polyp*” or “rect* cancer” or “rect* tumor*” or “rect* tumour*” or “rect* oncolog*” or “rect* carcinoma*” or “rect* mass*” or “rect* malignan*” or “neoplas* rect*” or “rect* neoplas*” or “anal cancer*” or “anal carcinoma*” or “anal mass*” or “anus cancer*” or “anus carcinoma*”) 19. Limit 18 to yr=“2019-Current” 20. 17 or 19 21. exp elective surgery/ 22. Limit 21 to yr=“2019-Current” 23. (“elective surgery” or “elective surgical procedure*” or “elective surg*” or “elective operation*” or “elective surg* intervention*” or “non-emergen* surgery” or “non-emergen* surg*” or “non-emergen* surgical procedure*” or “non-emergen* surg*” or “non-emergen* operation*” or “non-emergen* procedure*” or “elective procedure*” or “elective surg* intervention*” or “non-emergency surg* intervention*” or “non-emergen* operation*”) 24. Limit 23 to yr=“2019-Current” 25. 22 or 24 26. 5 and 10 and 25 27. 5 and 10 and 25 and 15 28. 5 and 10 and 25 and 20 29. 5 and 10 and 25 and 15 and 20 |
