# Supplementary material for: Global elective breast- and colorectal cancer surgery performance backlogs, attributable mortality and implemented health system responses during the COVID-19 pandemic: A scoping review
Source: PLOS Glob Public Health. 2023 Apr 4;3(4):e0001413. doi: 10.1371/journal.pgph.0001413 (PMC10072489; doi:10.1371/journal.pgph.0001413)
Supplement: S4 Table — (DOCX) [file pgph.0001413.s008.docx]

**S4 Table** – Overall survival outcomes for delayed elective colorectal cancer surgery during the COVID-19 pandemic

| **No.** | **Authors**  **(Year of publication)** | **Study design** | **Country** | **Number of studies included** | **Delay**  **(From diagnosis to elective surgery)**  **(weeks)** | **No. of datasets**  **(Patient number not specified)** | **Number needed to harm** | **Hazard ratio (95% CI) for overall survival** | ***p*-value** |
| --- | --- | --- | --- | --- | --- | --- | --- | --- | --- |
| 1. | Whittaker et al. (2021) | Systematic review and meta-analysis | U.K. | N=7 | 4 | n=6 | 35 | 1.13 (1.02 – 1.26) | 0.02 |
|  |  |  |  |  | 12 | n=3 | 10 | 1.57 (1.16 – 2.12) | 0.004 |
