# Supplementary material for: Global elective breast- and colorectal cancer surgery performance backlogs, attributable mortality and implemented health system responses during the COVID-19 pandemic: A scoping review
Source: PLOS Glob Public Health. 2023 Apr 4;3(4):e0001413. doi: 10.1371/journal.pgph.0001413 (PMC10072489; doi:10.1371/journal.pgph.0001413)
Supplement: S5 Table — (DOCX) [file pgph.0001413.s009.docx]

**S5 Table** – Structural health system responses for elective breast surgery delays

| **BREAST CANCER** | | | | **STRUCTURES: INFRASTRUCTURE, EQUIPMENT, RESOURCES AND GUIDELINES** | | | | | | | |
| --- | --- | --- | --- | --- | --- | --- | --- | --- | --- | --- | --- |
| **No.** | **Authors (Year of publication)** | **Study design** | **Country** | **SARS-CoV-2**  **screening facilities for patients and HCP** | **PPE stockage** | **Separate SARS-CoV-2 treatment pathways** | **Physical distancing of hospital beds** | **Adapt clinical guidelines** | **Thorough OT sanitation between procedures** | **Other** | **Description** |
| 1 | Fregatti et al. (2020) | Case series | Italy | **✓** | **✓** |  |  | **✓** |  |  |  |
| 2 | Pelle et al. (2020) | Case series | Italy | **✓** |  |  | **✓** |  |  |  |  |
| 3 | Philouze et al. (2020) | Review | France | **✓** | **✓** | **✓** |  |  |  | **✓** | - Decrease number of operational OTs (prevent contamination) |
| 4. | Tam et al. (2020) | Case series | U.K. | **✓** |  | **✓** |  |  |  | **✓** | - Designated area for doffing of PPE |
| 5. | Faulkner et al. (2022) | Case series | USA | **✓** | **✓** |  |  |  |  |  |  |
| 6. | Tzeng et al. (2020) | Review | USA | **✓** | **✓** |  |  | **✓** |  |  |  |
| 7. | Nekkanti et al. (2020) | Case series | India | **✓** | **✓** | **✓** |  |  |  | **✓** | - Safe waste disposal |
| 8. | Irukulla et al. (2020) | Review | India | **✓** |  | **✓** |  | **✓** |  | **✓** | - Augment stocks of oncologic drugs for interim medical therapy - Decentralise chemotherapy due to travel ban |
| 9. | Leite et al. (2020) | Cohort study | Brazil | **✓** | **✓** | **✓** |  | **✓** |  |  |  |
| 10. | Aguiar et al. (2020) | Cross-sectional | Brazil | **✓** |  |  |  |  |  |  |  |
