# Supplementary material for: Global elective breast- and colorectal cancer surgery performance backlogs, attributable mortality and implemented health system responses during the COVID-19 pandemic: A scoping review
Source: PLOS Glob Public Health. 2023 Apr 4;3(4):e0001413. doi: 10.1371/journal.pgph.0001413 (PMC10072489; doi:10.1371/journal.pgph.0001413)
Supplement: S6 Table — (DOCX) [file pgph.0001413.s010.docx]

| **No.** | **Authors (Year of publication)** | **Study design** | **Country** | **SARS-CoV-2**  **screening facilities for patients and HCP** | **PPE stockage** | **Separate SARS-CoV-2 treatment pathways** | **Physical distancing of hospital beds** | **Negative-pressure ventilation within OTs** | **Smoke extraction equipment (laparoscopy)** | **Adapt clinical guidelines** | **OT sanitation**  **emphasis** | **Other** | **Description** |
| --- | --- | --- | --- | --- | --- | --- | --- | --- | --- | --- | --- | --- | --- |
| 1 | Pertile et al. (2020) | Case series | Italy | **✓** |  |  |  |  |  |  |  |  |  |
| 2 | Di Marzo et al. (2020) | Case series | Italy | **✓** |  |  |  | **✓** | **✓** |  |  |  |  |
| 3 | Evans et al. (2020) | Review | U.K. | **✓** |  | **✓** | **✓** |  |  |  | **✓** |  |  |
| 4 | Huddy et al. (2021a, b) | Case series | U.K. | **✓** |  | **✓** | **✓** |  | **✓** |  | **✓** | **✓** | - Robotic surgery - Maintain database of surgical patients |
| 5 | Carvalho et al (2022) | Case series | U.K. | **✓** | **✓** | **✓** | **✓** | **✓** | **✓** | **✓** |  |  |  |
| 6 | Conefrey et al (2022) | Qualitative study | U.K. | **✓** | **✓** | **✓** |  |  |  |  |  | **✓** | - Relocate surgery to private sector |
| 7 | Akbulut et al (2022) | Review | Turkey | **✓** |  |  |  |  |  | **✓** |  | **✓** | - Less invasive screening protocol |
| 8 | Jiang & Ma (2021) | Review | China | **✓** |  | **✓** | **✓** | **✓** |  |  | **✓** | **✓** | - Safe waste disposal |
| 9 | Nunoo-Mensah et al (2020) | Case series | Global | **✓** | **✓** |  | **✓** |  |  | **✓** |  |  |  |
| **BREAST- OR COLORECTAL CANCER** | | | | | | | | | | | | | |
| 1 | Balla et al. (2021) | Case-control study | Italy | **✓** |  | **✓** |  |  | **✓** |  |  | **✓** | - Hospital map - Designated area for doffing of PPE |
| 2 | Al-Jabir et al. (2020) | Review | U.K. | **✓** |  | **✓** |  |  | **✓** | **✓** |  | **✓** | - Adapt surgical consent (minimised in-person contact) |
| 3 | Glasbey et al. (2021) | Cohort study | Global: 55 countries | **✓** |  | **✓** | **✓** |  |  |  |  |  |  |
| 4 | COVIDSurg Collab (2020) | Review | Global: 11 Regions | **✓** | **✓** |  | **✓** |  |  |  |  | **✓** | - Plan for pandemic preparedness |
| 5 | Moletta et al. (2020) | Systematic review | Global incl. U.K. | **✓** | **✓** |  |  | **✓** | **✓** | **✓** | **✓** | **✓** | - Discard soda lime post procedures |

**S6 Table** – Further structural health system responses for elective breast- or colorectal cancer surgery
