# Supplementary material for: Global elective breast- and colorectal cancer surgery performance backlogs, attributable mortality and implemented health system responses during the COVID-19 pandemic: A scoping review
Source: PLOS Glob Public Health. 2023 Apr 4;3(4):e0001413. doi: 10.1371/journal.pgph.0001413 (PMC10072489; doi:10.1371/journal.pgph.0001413)
Supplement: S7 Table — (DOCX) [file pgph.0001413.s011.docx]

**S7 Table** – Health policy responses for elective breast cancer surgery delays

| **BREAST CANCER** | | | | **STRUCTURES**: **POLICY** | | | | | |
| --- | --- | --- | --- | --- | --- | --- | --- | --- | --- |
| **No.** | **Authors (Year of publication)** | **Study design** | **Country** | **Oncologic surgery services not suspended** | **Prohibit hospital visitors** | **Restrict public access to hospital areas** | **Suspended screening and diagnostic tests** | **Other** | **Description** |
| 1 | Fregatti et al. (2020) | Case series | Italy |  | **✓** |  |  |  |  |
| 2 | Pelle et al. (2020) | Case series | Italy |  | **✓** | **✓** |  |  |  |
| 3 | Philouze et al. (2020) | Review | France | **✓** |  |  |  |  |  |
| 4 | Tam et al. (2020) | Case series | U.K. | **✓** | **✓** |  |  |  |  |
| 5 | Tzeng et al. (2020) | Review | USA |  |  |  |  | **✓** | - Mandatory quarantine for out-of-state patients traveling to healthcare facilities |
| 6 | Nekkanti et al. (2020) | Case series | India |  |  |  |  |  |  |
| 7 | Irukulla et al. (2020) | Review | India |  | **✓** |  | **✓** |  |  |
| 8 | Leite et al. (2020) | Cohort study | Brazil | **✓** |  |  |  | **✓** | - Mandatory influenza vaccination or facemask usage for HCP |
| 9 | Aguiar et al. (2020) | Cross-sectional | Brazil |  |  |  |  |  |  |
