# Supplementary material for: Global elective breast- and colorectal cancer surgery performance backlogs, attributable mortality and implemented health system responses during the COVID-19 pandemic: A scoping review
Source: PLOS Glob Public Health. 2023 Apr 4;3(4):e0001413. doi: 10.1371/journal.pgph.0001413 (PMC10072489; doi:10.1371/journal.pgph.0001413)
Supplement: S8 Table — (DOCX) [file pgph.0001413.s012.docx]

**S8 Table** – Health policy responses for elective breast- and colorectal cancer surgery delays

| **COLORECTAL CANCER** | | | | **STRUCTURES: POLICY** | | | | | |
| --- | --- | --- | --- | --- | --- | --- | --- | --- | --- |
| **No.** | **Authors (Year of publication)** | **Study design** | **Country** | **Oncologic surgery services not suspended** | **Prohibit hospital visitors** | **Restrict public access to hospital areas** | **Restrict max. no. of cases performed per day** | **Suspended screening and diagnostic tests** | **Mandatory influenza vaccination or facemask usage for HCP** |
| 1 | Pertile et al. (2020) | Case series | Italy |  | **✓** | **✓** |  |  |  |
| 2 | Di Marzo et al. (2020) | Case series | Italy |  |  |  |  |  |  |
| 3 | Evans et al. (2020) | Review | U.K. |  | **✓** |  |  |  |  |
| 4 | Huddy et al. (2021) | Case series | U.K. | **✓** | **✓** |  | **✓** |  |  |
| 5 | Jiang and Ma (2021) | Review | China |  |  |  |  |  |  |
| 6 | Nunoo-Mensah et al. (2020) | Case series | Global |  |  |  |  | **✓** |  |
| **BREAST- OR COLORECTAL CANCER** | | | | | | | | | |
| 1 | Balla et al. (2021) | Case-control study | Italy | **✓** | **✓** |  |  |  |  |
| 2 | Al-Jabir et al. (2020) | Review | U.K. |  |  |  |  |  |  |
| 3 | Glasbey et al. (2021) | Cohort study | Global: 55 countries |  |  |  |  |  |  |
| 4 | Moletta et al. (2020) | Systematic review | Global incl. U.K. |  |  |  |  |  |  |
