# Supplementary material for: Global elective breast- and colorectal cancer surgery performance backlogs, attributable mortality and implemented health system responses during the COVID-19 pandemic: A scoping review
Source: PLOS Glob Public Health. 2023 Apr 4;3(4):e0001413. doi: 10.1371/journal.pgph.0001413 (PMC10072489; doi:10.1371/journal.pgph.0001413)
Supplement: S9 Table — (DOCX) [file pgph.0001413.s013.docx]

**S9 Table** – Human resources responses for elective breast cancer surgery delays

| **BREAST CANCER** | | | | **STRUCTURES: ORGANISATION OF HUMAN RESOURCES** | | | | | | |
| --- | --- | --- | --- | --- | --- | --- | --- | --- | --- | --- |
| **No.** | **Authors (Year of publication)** | **Study design** | **Country** | **Multidisciplinary collaboration** | **Dedicated medical teams for COVID-19 units** | **Clinical decision-making and procedures delegated to most experienced clinicians (maximise efficiency)** | **Minimise number of HCP in OT** | **Training** | **Other** | **Description** |
| 1 | Fregatti et al. (2020) | Case series | Italy |  |  |  |  | **✓** |  | - HCP training on COVID-19 mitigation |
| 2 | Pelle et al. (2020) | Case series | Italy | **✓** |  |  | **✓** |  |  |  |
| 3 | Philouze et al. (2020) | Review | France | **✓** |  | **✓** |  | **✓** |  | - HCP training by hospital hygiene team |
| 4 | Tam et al. (2020) | Case series | U.K. |  | **✓** |  |  | **✓** | **✓** | - Dedicated medical teams for COVID-19 units |
| 5 | Tzeng et al. (2020) | Review | USA |  |  |  |  |  |  |  |
| 6 | Nekkanti et al. (2020) | Case series | India |  |  |  |  |  |  |  |
| 7 | Irukulla et al. (2020) | Review | India |  |  |  |  |  |  |  |
| 8 | Leite et al. (2020) | Cohort study | Brazil | **✓** |  |  | **✓** |  |  |  |
| 9 | Aguiar et al. (2020) | Cross-sectional | Brazil |  |  |  |  |  |  |  |
| 10 | Nagarkar et al (2021) | Case series | India |  |  |  |  |  | **✓** | - Mental wellbeing support services |
