# Supplementary material for: Global elective breast- and colorectal cancer surgery performance backlogs, attributable mortality and implemented health system responses during the COVID-19 pandemic: A scoping review
Source: PLOS Glob Public Health. 2023 Apr 4;3(4):e0001413. doi: 10.1371/journal.pgph.0001413 (PMC10072489; doi:10.1371/journal.pgph.0001413)
Supplement: S10 Table — (DOCX) [file pgph.0001413.s014.docx]

**S10 Table** – Human resources responses for elective colorectal cancer surgery delays

| **COLORECTAL CANCER** | | | | **STRUCTURES: ORGANISATION OF HUMAN RESOURCES** | | | | | | |
| --- | --- | --- | --- | --- | --- | --- | --- | --- | --- | --- |
| **No.** | **Authors (Year of publication)** | **Study design** | **Country** | **Multidisciplinary collaboration** | **Dedicated medical teams for COVID-19 units** | **Clinical decision-making and procedures delegated to most experienced clinicians (maximise efficiency)** | **Minimise number of HCP in OT** | **Training** | **Other** | **Description** |
| 1 | Pertile et al. (2020) | Case series | Italy | **✓** |  |  |  | **✓** | **✓** | - Nurses trained to provide home-based post-operative care - HCP allocated to accompany patients with disability |
| 2 | Di Marzo et al. (2020) | Case series | Italy |  |  |  |  |  |  |  |
| 3 | Evans et al. (2020) | Review | U.K. |  |  | **✓** |  |  |  |  |
| 4 | Huddy et al. (2021) | Case series | U.K. |  |  | **✓** |  |  | **✓** | - Staff handover of patients’ food in airlocked corridors (prevent contamination) |
| 5 | Jiang and Ma (2021) | Review | China | **✓** |  |  |  |  |  |  |
| 6 | Nunoo-Mensah et al. (2020) | Case series | Global |  |  |  |  |  |  |  |
| 7 | Balla et al. (2021) | Case-control study | Italy |  |  |  | **✓** | **✓** |  |  |
| 8 | Al-Jabir et al. (2020) | Review | U.K. |  |  |  |  |  |  |  |
| 9 | Glasbey et al. (2021) | Cohort study | Global: 55 countries | **✓** |  |  | **✓** |  | **✓** | - Staff to accompany patients’ hospital navigation |
| 10 | Moletta et al. (2020) | Systematic review | Global incl. U.K. |  |  | **✓** |  |  | **✓** | - Change scrubs, shower post-surgery |
