# Supplementary material for: Global elective breast- and colorectal cancer surgery performance backlogs, attributable mortality and implemented health system responses during the COVID-19 pandemic: A scoping review
Source: PLOS Glob Public Health. 2023 Apr 4;3(4):e0001413. doi: 10.1371/journal.pgph.0001413 (PMC10072489; doi:10.1371/journal.pgph.0001413)
Supplement: S11 Table — (DOCX) [file pgph.0001413.s015.docx]

**S11 Table** – Adapted healthcare provision processes for elective breast cancer surgery delays

| **BREAST CANCER** | | | | **PROCESSES: HEALTHCARE PROVISION** | | | | | | | | |
| --- | --- | --- | --- | --- | --- | --- | --- | --- | --- | --- | --- | --- |
| **No.** | **Authors (Year of publication)** | **Study design** | **Country** | **Telehealth utilisation** | **Clinical case prioritisation and triage** | **Interim medical therapy: NACRT** | **Expedited discharge** | **Infection prevention and control measures: PPE, hand hygiene** | **SARS-CoV-2 infection – Disease notification** | **Decreased frequency of health consultations** | **Other** | **Description** |
| 1 | Fregatti et al. (2020) | Case series | Italy | **✓** | **✓** |  |  |  | **✓** | **✓** |  |  |
| 2 | Pelle et al. (2020) | Case series | Italy | **✓** | **✓** |  | **✓** | **✓** |  |  |  |  |
| 3 | Philouze et al. (2020) | Review | France |  | **✓** | **✓** |  |  |  |  |  |  |
| 4 | Tam et al. (2020) | Case series | U.K. |  |  |  | **✓** |  |  |  | **✓** | - Household contacts to quarantine pre- and post-surgery |
| 5 | Faulkner et al. (2022) | Case series | USA |  | **✓** | **✓** | **✓** |  |  |  |  |  |
| 6 | Tzeng et al. (2020) | Review | USA |  | **✓** | **✓** |  |  |  |  |  |  |
| 7 | Nekkanti et al. (2020) | Case series | India |  |  |  |  | **✓** |  |  | **✓** | - Detected SARS-CoV-2 infection pre-operatively |
| 8 | Irukulla et al. (2020) | Review | India | **✓** | **✓** | **✓** |  |  |  |  |  |  |
| 9 | Leite et al. (2020) | Cohort study | Brazil | **✓** |  |  |  | **✓** |  |  |  |  |
| 10 | Aguiar et al. (2020) | Cross-sectional | Brazil |  |  |  |  |  |  |  |  |  |
