# Supplementary material for: Global elective breast- and colorectal cancer surgery performance backlogs, attributable mortality and implemented health system responses during the COVID-19 pandemic: A scoping review
Source: PLOS Glob Public Health. 2023 Apr 4;3(4):e0001413. doi: 10.1371/journal.pgph.0001413 (PMC10072489; doi:10.1371/journal.pgph.0001413)
Supplement: S12 Table — (DOCX) [file pgph.0001413.s016.docx]

**S12 Table** – Adapted healthcare provision processes for elective breast- and colorectal cancer surgery delays

| **COLORECTAL CANCER** | | | | **PROCESSES: HEALTHCARE PROVISION** | | | | | | | | |
| --- | --- | --- | --- | --- | --- | --- | --- | --- | --- | --- | --- | --- |
| **No.** | **Authors (Year of publication)** | **Study design** | **Country** | **Telehealth or digital apps** | **Clinical case prioritisation and triage** | **Interim medical therapy:**  **NACRT** | **Expedited discharge** | **Infection prevention & control: PPE, hand hygiene** | **SARS-CoV-2 infection – Disease notification** | **Decreased frequency of health consultations** | **Other** | **Description** |
| 1 | Pertile et al. (2020) | Case series | Italy | **✓** | **✓** | **✓** | **✓** | **✓** | **✓** |  | **✓** | - Update relatives telephonically |
| 2 | Di Marzo et al. (2020) | Case series | Italy | **✓** |  | **✓** |  |  |  |  | **✓** | - ‘Watch-and-wait’ approach |
| 3 | Evans et al. (2020) | Review | U.K. |  | **✓** |  |  | **✓** |  |  |  |  |
| 4 | Huddy et al. (2021) | Case series | U.K. |  |  |  |  |  |  |  | **✓** | - Robotic surgery |
| 5 | Jiang and Ma (2021) | Review | China | **✓** |  |  | **✓** | **✓** | **✓** |  |  |  |
| 6 | Raj Kumar et al (2020) | Case series | India | **✓** |  | **✓** |  | **✓** |  | **✓** |  |  |
| 7 | Ozdemir and Temiz (2021) | Case series | Turkey |  | **✓** | **✓** |  | **✓** |  |  |  |  |
| 8 | Akbulut et al (2022) | Review | Turkey | **✓** | **✓** | **✓** |  |  |  | **✓** | **✓** | - Non-aerosolising, non-invasive screening |
| 9 | COVIDSurg Collab (2020) | Review | Global: 11 Regions |  |  |  |  | **✓** |  | **✓** | **✓** | - Team-based surgical care during COVID-19 |
| 10 | Nunoo-Mensah et al. (2020) | Case series | Global | **✓** | **✓** | **✓** |  | **✓** |  |  | **✓** | - Minimally invasive surgery - Defer NACRT during SARS-CoV-2 upsurges |
| **BREAST- OR COLORECTAL CANCER** | | | | | | | | | | | | |
| 1 | Balla et al. (2021) | Case-control | Italy |  |  |  |  | **✓** |  |  | **✓** | - Minimally invasive surgery |
| 2 | Al-Jabir et al. (2020) | Review | U.K. | **✓** | **✓** | **✓** | **✓** |  |  | **✓** | **✓** | - Breast conserving surgery - Defer NACRT during SARS-CoV-2 upsurges |
| 4 | Nagarkar et al. (2021) | Case series | India | **✓** | **✓** |  |  | **✓** |  | **✓** | **✓** | - Informed consent (SARS-CoV-2 risk exposure) |
| 5 | Glasbey et al. (2021) | Cohort | Global: 55 countries | **✓** | **✓** |  |  | **✓** |  |  |  | - Defer laparoscopy for patients with COVID-19 |
| 6 | Moletta et al. (2020) | Systematic review | Global incl. U.K. |  |  | **✓** |  |  |  |  | **✓** | - HCP shower post-surgery - Regard every patient as SARS-CoV-2-infected until proven otherwise |
