# Supplementary material for: Global elective breast- and colorectal cancer surgery performance backlogs, attributable mortality and implemented health system responses during the COVID-19 pandemic: A scoping review
Source: PLOS Glob Public Health. 2023 Apr 4;3(4):e0001413. doi: 10.1371/journal.pgph.0001413 (PMC10072489; doi:10.1371/journal.pgph.0001413)
Supplement: S13 Table — (DOCX) [file pgph.0001413.s017.docx]

**S13 Table** – Clinical outcomes as health system response efficacy indicators for delays in elective breast cancer surgery

| **BREAST CANCER** | | | | **OUTCOMES: METRICS OF HEALTH SYSTEM RESPONSE EFFICACY** | | | | | | | | |
| --- | --- | --- | --- | --- | --- | --- | --- | --- | --- | --- | --- | --- |
| **No.** | **Authors (Year of publication)** | **Study design** | **Country** | **Postoperative SARS-CoV-2 infection (patients)** | **SARS-CoV-2 infection (HCP)** | **Number of procedures performed without delay** | **Length of hospital stay** | **Hospital readmission** | **Postoperative complications (incl. pulmonary complications)** | **Waiting time for surgery (days)** | **Other** | **Description** |
| 1 | Fregatti et al. (2020) | Case series | Italy | **✓** |  | **✓** | **✓** | **✓** |  |  |  |  |
| 2 | Pelle et al. (2020) | Case series | Italy |  | **✓** |  |  | **✓** | **✓** |  |  |  |
| 3 | Philouze et al. (2020) | Review | France | **✓** | **✓** | **✓** |  |  | **✓** |  | **✓** | - Postoperative SARS-CoV-2-attributable mortality |
| 4 | Javed et al (2021) | Case series | U.K. | **✓** |  |  |  |  | **✓** |  |  |  |
| 5 | Tam et al. (2020) | Case series | U.K. | **✓** |  |  |  |  | **✓** | **✓** | **✓** | - Postoperative mortality |
| 6 | Faulkner et al (2021) | Case series | USA | **✓** |  |  | **✓** |  | **✓** |  |  |  |
| 7 | Tzeng et al. (2020) | Review | USA |  |  |  |  |  |  |  |  |  |
| 8 | Nekkanti et al. (2020) | Case series | India |  |  | **✓** |  |  | **✓** |  | **✓** | - Postoperative SARS-CoV-2-attributable morbidity - Number of asymptomatic infections detected on pre-operative SARS-CoV-2 screening |
| 8 | Irukulla et al. (2020) | Review | India |  |  |  |  |  |  |  |  |  |
| 9 | Leite et al. (2020) | Cohort study | Brazil | **✓** |  | **✓** |  |  |  |  |  |  |
| 10 | Aguiar et al. (2020) | Cross-sectional | Brazil | **✓** |  | **✓** |  | **✓** |  |  |  |  |
