# Supplementary material for: Global elective breast- and colorectal cancer surgery performance backlogs, attributable mortality and implemented health system responses during the COVID-19 pandemic: A scoping review
Source: PLOS Glob Public Health. 2023 Apr 4;3(4):e0001413. doi: 10.1371/journal.pgph.0001413 (PMC10072489; doi:10.1371/journal.pgph.0001413)
Supplement: S14 Table — (DOCX) [file pgph.0001413.s018.docx]

**S14 Table** – Clinical outcomes as health system response efficacy indicators for delays in elective breast- and colorectal cancer surgery

| **COLORECTAL CANCER** | | | | **OUTCOMES: METRICS OF HEALTH SYSTEM RESPONSE EFFICACY** | | | | | | | | |
| --- | --- | --- | --- | --- | --- | --- | --- | --- | --- | --- | --- | --- |
| **No.** | **Authors (Year of publication)** | **Study design** | **Country** | **Postoperative SARS-CoV-2 infection (patients)** | **SARS-CoV-2 infection (HCP)** | **Number of procedures performed without delay** | **Length of hospital stay** | **Hospital readmission** | **Postoperative complications (incl. pulmonary complications)** | **Waiting time for surgery (days)** | **Other** | **Description** |
| 1 | Pertile et al. (2020) | Case series | Italy |  |  |  | **✓** |  | **✓** | **✓** |  |  |
| 2 | Di Marzo et al. (2020) | Case series | Italy |  |  |  |  |  |  |  |  |  |
| 3 | Evans et al. (2020) | Review | U.K. |  | **✓** | **✓** |  | **✓** | **✓** |  | **✓** | - Histologic tumour upstaging |
| 4 | Huddy et al. (2021) | Case series | U.K. |  | **✓** | **✓** | **✓** | **✓** | **✓** |  | **✓** | - Robotic surgery pros & cons - Histologic tumour upstaging - Inpatient SARS-CoV-2 infection |
| 5 | Carvalho et al (2022) | Case series | U.K. | **✓** |  |  |  | **✓** | **✓** |  | **✓** | - 30-day mortality post surgery |
| 6 | Feier et al (2022) | Review | Romania |  |  |  | **✓** |  |  |  |  |  |
| 7 | Ozdemir and Temiz (2021) | Case series | Turkey | **✓** |  |  |  |  | **✓** |  |  |  |
| 8 | Jiang and Ma (2021) | Review | China |  |  | **✓** | **✓** |  | **✓** |  | **✓** | - Histologic tumour upstaging |
| 9 | Nunoo-Mensah et al. (2020) | Case series | Global |  |  |  |  |  |  |  |  |  |
| **BREAST- OR COLORECTAL CANCER** | | | | | | | | | | | | |
| 1 | Balla et al. (2021) | Case-control study | Italy |  | **✓** |  |  |  |  |  |  |  |
| 2 | Al-Jabir et al. (2020) | Review | U.K. |  |  |  |  |  |  |  |  |  |
| 3 | Kronenfield et al (2021) | Case series | USA | **✓** |  |  |  |  | **✓** |  | **✓** | 30-day (postoperative) incidence of COVID-19 |
| 4 | Glasbey et al. (2021) | Cohort study | Global: 55 countries | **✓** |  | **✓** |  |  | **✓** |  |  |  |
| 5 | Moletta et al. (2020) | Systematic review | Global incl. U.K. |  | **✓** | **✓** |  |  |  |  |  |  |
